# Intracellular Nanopharmacology of Trastuzumab Deruxtecan Reveals Lysosome-Centered Organelle Vulnerabilities in HER2-Positive Breast Cancer Cells

**DOI:** 10.1021/acsnanomed.6c00097

**Published:** 2026-07-02

**Authors:** Erica Tagliatti, Maria Cristina Gagliani, Grazia Bellese, Shahnaz Salamat, Martina Crippa, Manuela Sollazzo, Andrea Abbona, Matteo Paccagnella, Pietro Arnaldi, Lucilla Rossi, Andrea Petretto, Paola Rusmini, Valeria Crippa, Marco Carlo Merlano, Ornella Garrone, Anna Maria Porcelli, Michela Matteoli, Paola Falletta, Patrizio Castagnola, Katia Cortese

**Affiliations:** 1 Laboratory of Pharmacology and Brain Pathology, IRCCS Humanitas Research Hospital, Via Alessandro Manzoni 56, 20089 Rozzano, Milan, Italy; 2 DIMES, Department of Experimental Medicine, MorphoLAB, 9302 University of Genoa, Via Antonio De Toni 14, 16132 Genoa, Italy; 3 Experimental Imaging Center, IRCCS Ospedale San Raffaele, Via Olgettina 60, 20132 Milan, Italy; 4 FABIT, Department of Pharmacy and Biotechnology, University of Bologna, Via Zamboni, 33, 40126 Bologna, Italy; 5 Fondazione Arco, Via Antonio Carle, 25, 12100 Cuneo, Italy; 6 Azienda Ospedaliera Santa Croce e Carle, Via Michele Coppino 26, 12100 Cuneo, Italy; 7 Core Facility for Omics Sciences, 18572 IRCCS Istituto Giannina Gaslini, Via Gerolamo Gaslini 5, 16147 Genoa, Italy; 8 Dipartimento di Scienze Farmacologiche e Biomolecolari “Rodolfo Paoletti”, Dipartimento di Eccellenza 2018-2027, 9304 Università degli Studi di Milano, Via Giuseppe Balzaretti 9, 20133 Milan, Italy; 9 Scientific Direction, Candiolo Cancer Institute, FPO-IRCCS, Viale della Ricerca 7, 10060 Candiolo, Italy; 10 Medical Oncology, Fondazione IRCCS Ca’ Granda Ospedale Maggiore Policlinico, Via Francesco Sforza 28, 20122 Milano, Italy; 11 FABIT, Department of Pharmacy and Biotechnology and CIRI, Interdepartmental Centre for Industrial Research ’Scienze Della Vita e Tecnologie per La Salute’, University of Bologna, Via Zamboni 33, 40126 Bologna, Italy; 12 IRCCS Azienda Ospedaliero-Universitaria di Bologna, Via Giuseppe Massarenti 9, 40138 Bologna, Italy; 13 CNR Institute of Neuroscience, Via Raoul Follereau 3, 20854 Milano, Italy; 14 9372 Vita-Salute San Raffaele University, Via Olgettina 60, 20132 Milan, Italy; 15 IRCCS Azienda Ospedaliera Metropolitana, Largo Rosanna Benzi 10, 16132 Genova, Italy

**Keywords:** trastuzumab deruxtecan (T-DXd), antibody−drug conjugates, nanoscale drug trafficking, lysosomal remodeling, electron microscopy, HER2-positive breast cancer

## Abstract

Trastuzumab-deruxtecan (T-DXd) is a clinically effective antibody–drug conjugate (ADC) with activity across HER2-amplified, HER2-low, and ultralow breast cancers. Despite its clinical success, how its nanoscale intracellular trafficking and processing shape therapeutic outcomes remains incompletely defined. Given the central role of HER2 internalization and lysosomal processing in ADC pharmacology, we investigated the spatiotemporal intracellular response to T-DXd in human HER2-positive breast cancer cell models. By integrating biochemical analyses with light and electron microscopy-based nanoscale imaging, and employing orthogonal nanoscale probes, including BSA–gold nanoparticles and nanogold-based labeling, together with proteomic profiling, we reconstructed the temporal sequence of T-DXd action over 72 h. An early phase (2–24 h) was characterized by rapid HER2 phosphorylation, sustained ERK signaling, metabolic activation, and TFEB-driven lysosomal engagement, consistent with active drug processing. Nanoscale probing of the endocytic pathway using BSA–gold nanoparticles demonstrated a marked expansion of the lysosomal compartment, with increased lysosome number and size, supporting the concept that T-DXd actively remodels lysosomal architecture rather then passively exploiting it as a delivery site. A transitional phase at 48 h revealed pronounced lysosomal accumulation of T-DXd and extensive organelle remodeling. By 72 h, cells entered a late phase marked by mitochondrial dysfunction, nuclear envelope stress, and DNA damage, accompanied by the emergence of nanoscale contacts among lysosomes, mitochondria, and the nucleus, suggestive of coordinated organelle failure. Proteomic analysis indicated activation of inflammatory and stress-associated pathways, including TNFα/NF-κB signaling, supported by increased release of IL-6, IL-8, and TNF-α. Collectively, this spatiotemporal framework identifies compartment-specific nanoscale vulnerabilities engaged by T-DXd and highlights ERK-dependent signaling and lysosomal function as potential targets for rational combination strategies in HER2-positive breast cancer.

## Graphical abstract

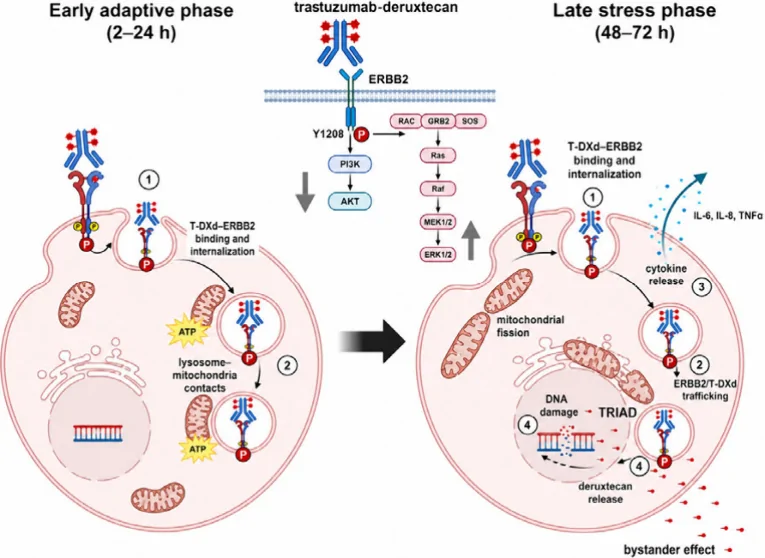

## Introduction

Breast cancer (BCa) is a heterogeneous disease classically stratified by the expression of estrogen (ER), progesterone (PgR) receptors, and the human epidermal growth factor receptor 2 (HER2/ERBB2). Approximately 15–20% of tumors overexpress HER2, which has historically been associated with aggressive behavior and poor prognosis.^1^ Over the past two decades, the clinical management of HER2-positive breast cancer has been transformed by HER2-targeted agents, including monoclonal antibodies such as trastuzumab and pertuzumab, tyrosine kinase inhibitors such as lapatinib, neratinib, and tucatinib, and antibody–drug conjugates (ADCs).^2,3^ Among these, trastuzumab deruxtecan (T-DXd, DS-8201a) has emerged as a highly effective HER2-targeting ADC owing to its cleavable linker, membrane-permeable deruxtecan (DXd) payload, bystander activity, and improved drug delivery properties compared with earlier-generation ADCs such as trastuzumab emtansine (T-DM1/Kadcyla).^4–6^ T-DXd enables efficient internalization, lysosomal processing, and bystander killing of neighboring cells.^7,8^ Landmark DESTINY trials demonstrated robust activity not only in HER2-amplified tumors but also in HER2-low and ultralow diseases,^9–14^ highlighting that HER2 abundance alone does not fully predict therapeutic response.^15,16^ Additional studies have shown that T-DXd cytotoxicity in HER2-low and even HER2-negative contexts may occur independently of direct tumor-cell HER2 engagement through mechanisms including extracellular cathepsin-mediated linker cleavage, bystander killing, immunogenic activation, and FcγR-mediated processing by tumor-associated macrophages.^17,18^ The recent pan-tumor approval of T-DXd further emphasizes its relevance across diverse cancer types.^19–21^

Mechanistically, HER2 signals through ERBB heterodimerization and activates PI3K/AKT/mTOR and MAPK/ERK cascades while maintaining prolonged membrane residency due to inefficient endocytosis.^22–26^ However, previous work—including studies from our group—demonstrated that HER2 trafficking is highly plastic: HSP90 inhibition, HER2 kinase inhibition (neratinib), and trastuzumab treatment promote HER2 internalization, lysosomal engagement, and in some context’s autophagy, lipid and EV-associated remodeling.^27–34^ These findings reinforce the concept that HER2 is a trafficking-prone receptor whose intracellular routing can be reshaped by targeted interventions.^35^ Indeed, recent mechanistic work showed that ERBB2 internalization, Rab GTPase–regulated sorting, and recycling differentially influence the intracellular processing of distinct ADCs, including T-DM1 and T-DXd.^36^

Resistance mechanisms to HER2-directed therapies have also been delineated across molecular and genomic layers. PI3K/PTEN pathway activation is a central driver of trastuzumab resistance,^37,38^ while analyses of paired pre- and post-T-DXd patient samples revealed that nearly half of progressing tumors downregulate or completely lose HER2, impairing ADC binding and internalization.^11,35^ Additional regulators such as histamine *N*-methyltransferase (HNMT) modulate HER2 abundance, nuclear HER2 signaling, and ADC sensitivity in HER2-low or triple-negative contexts. Collectively, these observations suggest that determinants of T-DXd efficacy reside not solely at the plasma membrane, but within intracellular architectures and trafficking states as well. Consistent with this view, our recent characterization of HER2-positive 3D spheroids revealed that spatial constraints generate emergent organelle architectures not observed in 2D cultures, highlighting the value of morphological approaches to capture spatially organized cellular states.^39^ In line with this perspective, recent work has emphasized that nanomedicine-based strategies for HER2-positive breast cancer, including ADCs, are shaped not only by molecular target expression but also by tumor architecture, intracellular trafficking, organelle organization, and spatially constrained drug response.^40^ This aligns with the broader recognition that lysosomes act not simply as degradative end points but as dynamic hubs integrating metabolic, transcriptional, and stress responses.^41–43^

In this context, ADC activity is increasingly understood to unfold through time-dependent, compartmentalized, and interconnected subcellular events, rather than through a linear binding–internalization–degradation sequence. Based on these considerations, we hypothesized that T-DXd triggers a structured sequence of intracellular responses in HER2-positive cells. Here, we map the spatiotemporal progression of T-DXd activity using integrated nanoscale imaging, biochemical and proteomic analyses. We identify an early phase characterized by rapid signaling and progressive lysosomal engagement (2–24 h), a transitional “turning point” associated with lysosomal saturation and organelle remodeling (24–48 h), and a late phase marked by nuclear stress and loss of cellular integrity (72 h). This temporal framework provides a morphological perspective on the intracellular vulnerabilities that shape ADC efficacy.

## Materials And Methods

### Cell Culture and Treatments

HER2-overexpressing SKBR-3, BT474 and MDA-MB-361, were obtained from the Cell Factory of IRCCS Ospedale Policlinico San Martino (member of the European Culture Collection). Cells were cultured in high-glucose DMEM with 10% heat-inactivated FBS, 1% glutamine, and antibiotics (Euroclone) at 37 °C, 5% CO_2_. T-DXd (Enhertu, Daiichi Sankyo) was provided by Ospedale Ca’ Granda and dissolved in 0.9% NaCl (stock: 20 mg/mL). Trastuzumab (Tz, Genentech-Roche) was obtained from the UFA unit at IRCCS San Martino (stock: 21 mg/mL). Treatments were performed using Tz at 10 μg/mL for SKBR-3 and 0.21 μg/mL for BT474 cells, with matched IgG controls.^44^ T-DXd was applied at the same concentrations for direct comparison.

### Antibodies and Reagents

The following primary antibodies were used: Mouse monoclonal anti-HER2 (Ab-20, L87 + 2ERB19; Thermo Scientific, Waltham, MA, USA) for Western blot. Rabbit polyclonal anti-Phospho-HER2 (Y1248) (#2247, Cell Signaling Technology, Danvers, MA, USA). Mouse monoclonal anti-HER2 (9G6, Santa Cruz Biotechnology, Dallas, TX, USA) for EM and fluorescence microscopy. CoraLitePlus488-conjugated anti-HER2 monoclonal antibody (CL488-60311, Proteintech, USA) for live imaging. Pan-AKT (clone 40D4, #2920, Cell Signaling Technology) and p-AKT (Ser473, D9E XP, Cell Signaling Technology). p-AKT1/2/3 (Ser473, sc-7985-R, Santa Cruz). ERK1/2 (MK1, sc-135900, Santa Cruz) and p-ERK1/2 (#9101, Cell Signaling Technology). Mouse anti-LAMP1 (H4A3, Developmental Studies Hybridoma Bank, Iowa City, IA) for immunofluorescence. Rabbit monoclonal LAMP1 (D2D11, Cell Signaling Technology). Anti-SQSTM1/p62: Abnova (H00008878-M01) and Abcam (ab91526). Anti-LC3A/B: Novus Biologicals (NB100-2220) and Sigma-Aldrich (L8918). Anti-TFEB (A303-673A, Bethyl Laboratories). Anti-GAPDH (FL-335, sc-25778, Santa Cruz Biotechnology). Antivinculin (V9131, Sigma-Aldrich, used as loading control). Anti-Lamin B1: Abcam (ab16048) and Proteintech (12987-1-AP). Anti-β-actin: Invitrogen (15G5A11/E2) and Sigma-Aldrich (SAB5500001). Anti-4-hydroxynonenal (ab46545, Abcam). Anti-SOD2 (06-984, Sigma-Aldrich). Anti-PRDX3 (LF-PA0030, Thermo Fisher Scientific). Anticatalase (C0979, Sigma-Aldrich). HRP-conjugated goat antimouse IgG (1:5000; Jackson ImmunoResearch, 115-035-146). HRP-conjugated goat antirabbit IgG (1:5000; Jackson ImmunoResearch, 111-035-144). HRP-conjugation kit- Lighting link/ab102890, Abcam). 3,3′-diaminobenzidine tetrahydrochloride (DAB) SIGMAFAST DAB tablets (Sigma-Aldrich, Cat. No. D4418).

### Flow Cytometry (FCM) Analysis

Both adherent and floating cells were collected after 72 h of 10 μg/mL T-DXd treatment and centrifuged at 980*g* for 5 min. Apoptotic and necrotic cells were evaluated by using the Vybrant Apoptosis Assay Kit purchased from Thermo Fisher Scientific, Waltham, MA, USA, with a minor procedure modification as we used the nuclear staining fluorochrome Sytox Blue instead of the Sytox Green. Cells were then analyzed using a Beckman Coulter Cyan ADP flow cytometer (Beckman Coulter Life Sciences, Brea, CA, USA). Annexin-V-APC^+^/Sytox Blue^–^, Annexin-V-APC^+^/Sytox Blue^+^, and Annexin-V-APC^–^/Sytox Blue^+^ cells were identified as early apoptotic, late apoptotic and necrotic cells, respectively. An ANOVA with Tukey’s post-test was performed to assess statistical significance.

### Immunofluorescence Analyses

SKBR-3 cells were treated with 10 μg/mL T-DXd for 2 or 72 h, fixed in 3% PFA (PBS, pH 7.4, 20 min), and quenched with 30 mM NH_4_Cl. Cells were permeabilized with 0.2% saponin/0.1% BSA for 5 min. After PBS washes, Alexa Fluor 546 or 488-conjugated secondary antibodies (Thermo Fisher) were applied for 20 min at room temperature. Coverslips were mounted in ProLong Gold (Thermo Fisher) and imaged using an Olympus IX70 widefield microscope with Hamamatsu Orca-Flash 4.0 camera. Images were analyzed using Huygens Professional (SVI) with the object analyzer tool.

### Mitochondrial Morphology Analysis

Cells on coverslips were stained with 23.5 μM MitoTracker Red CMXRos and 5.9 μM Hoechst 33342 (Thermo Fisher) for 20 min at 37 °C, washed, and incubated for an additional 20 min in fresh medium. Fixation was done with 3.7% PFA/2% sucrose (in PBS, 5 min), followed by PBS washes and mounting. Imaging and real-time deconvolution were performed using a Zeiss Axio Imager A2M with Apotome module.

### Proliferation and Internalization Assay

To perform a proliferation and internalization screen in SKBR3, 100.000 cells were seeded overnight on a 24-well plate and treated with 10 μg/mL T-DXd and 5 μg/mL of CoraLitePlus488-HER2 Ab on the following day. Proliferation and internalization were measured for 72 h using the IncuCyteSX5 Live-Cell Analysis system (20x objective). Wells were imaged every 2 h (brightfield and green phase; acquisition time 300 ms). Live-cell image analysis was performed using IncuCyteSX5 software. Cell confluence was quantified from phase-contrast images using the IncuCyte “AI Confluence” segmentation module. The phase object was defined as “cells”; hole fill was set to 0.0000 μm^2^ and adjust size to 0 pixels. No additional filters were applied for area or eccentricity, which were retained at the software default value of 0.0000. Normalized proliferation was calculated using the IncuCyte metric “Cells Area Confluence Normalized to 0d0h0m”, corresponding to the relative change in confluence area compared with the initial time point. HER2-associated fluorescent puncta were quantified in the green channel using the IncuCyte metric “HER2 Coralite Count (per image)”. The green object was defined as “ERBB2 coralite” and segmented using the “Surface Fit” algorithm with a threshold of 2.0000 GCU and edge split enabled. Edge sensitivity was set to 0. Cleanup parameters were set as follows: hole fill = 0.0000 μm^2^ and adjust size = 0 pixels. No additional filtering thresholds were applied for area, eccentricity, mean intensity, or integrated intensity; minimum and maximum filter values were retained at 0.0000. To account for temporal changes in cell number and growth during treatment, HER2 Coralite Count per image was normalized to the corresponding cell confluence area obtained from the phase-contrast channel using the IncuCyte metric “Cells Area Confluence”. The normalized HER2-associated signal was calculated as HER2 Coralite Count per image divided by cell confluence area and then expressed relative to the initial time point.

### Immunoelectron Microscopy

#### Uptake of 5 nm BSA-Gold

After being washed with fresh medium, SKBR3 cells were incubated with BSA-gold (OD520 = 5) for 72 h and processed as described below.^28^ For lysosomes morphometric analysis, TEM images were acquired at 25,000× magnification. For each condition, 30 images were randomly selected from three independent experiments (*n* = 3). Lysosomes were identified based on the presence of internalized BSA–gold particles. Lysosomal size and number were quantified using EM Radius 2.0 software. Organelle contact sites were defined as membrane appositions with an interorganelle distance ≤30 nm. The frequency of lysosome–mitochondria contacts and lysosome–mitochondria–nucleus triads was quantified per cell profile.

#### T-DXd-HRP Conjugation

HRP conjugation of T-DXd was performed using the Lightning-Link HRP Conjugation Kit (ab102890, Abcam) following the manufacturer’s protocol. T-DXd was diluted from the stock solution (21 mg/mL) to a final concentration of 1 mg/mL in PBS before conjugation. The conjugated product was subsequently used for electron microscopy analyses.

#### T-DXd-HRP Internalization Assay

SKBR-3 cultured on glass chamber slides (Lab-Tek 177380, Nalge Nunc int., Rochester, NY, USA), were washed in CO2-independent medium (cat. number 18045070; Life Technologies) and incubated in CO_2_-independent medium containing 10 μg/mL T-DXd-HRP on ice for 30 min. After extensive washing with cold CO2-independent medium to remove unbound T-DXd-HRP, cells were shifted at 37 °C in CO_2_-independent medium for 24, 48 and 72 h. Internalization was ended as follows: cells were placed on ice in prechilled CO2-independent medium, incubated for 20 min at 4 °C in freshly prepared DAB buffer (1 mg/mL DAB and 0.012% H_2_O_2_), and fixed with 2.5% glutaraldehyde (Electron Microscopy Science, Hatfield, PA) for 1 h. The cells were subsequently processed for standard EM. Cells were postfixed in osmium tetroxide for 2 h and 1% uranyl acetate for 1 h at room temperature. Cells were next dehydrated through a graded ethanol series and flat embedded in resin (Poly-Bed; Polysciences, Inc., Warrington, PA, USA) for 24 h at 60 °C.

### NanoGold Labeling

for LAMP1-Positive Lysosomes in SKBR3 Cells. SKBR3 cells were fixed with 4% PFA (15–20 min), permeabilized with 0.1% saponin +0.1% BSA, and blocked (0.5% BSA, 50 mM NH_4_Cl, 0.1% saponin, 0.1% acetylated BSA, 150 mM NaCl, 30 min). Cells were incubated with a primary antibody against LAMP1 (1 h), followed by a Nanogold-conjugated secondary antibody (1 h). After washes, samples were postfixed in 2.5% glutaraldehyde and Nanogold was enhanced using sequential gold enhancement steps (10 min). Postfixation was performed with 1% OsO_4_ (1 h), followed by 1% uranyl acetate staining (30 min). Dehydration was carried out through graded EtOH, resin infiltration, and polymerization at 60 °C for 2 days before electron microscopy analysis. Ultrathin sections (50 nm) were cut parallel to the substrate, stained with 5% uranyl acetate in 50% ethanol and observed with a HITACHI 7800 120 Kv transmission electron microscope (Hitachi, Tokyo, Japan). Digital images were captured with a Megaview III camera.

### Immunoblot Analysis

For Western blotting, SKBR-3, BT474 and MDA-MB-361 cells were lysed using lysis buffer (Hepes pH 7.4 20 mM, NaCl 150 mM, 10% Glycerol, 1% Triton X-100) supplemented with protease inhibitors cocktail Complete (Roche Applied Science, Penzberg, Germany) and sodium orthovanadate and PhosStop (Roche, Roche Holding AG, Basel Switzerland). Proteins were resolved on SDS-polyacrylamide gel electrophoresis (Thermo Fisher Scientific Inc. Waltham, MA, USA) and blotted on nitrocellulose (GE Healthcare Life Science, Amersham Buckinghamshire, UK) or PVDF (Merck Millipore, Darmstadt, Germany) membranes. Detection was performed using ECL Detection Reagent (BIORAD, Hercules, CA, USA) according to manufacturer’s protocol. ECL signals were detected, recorded, and measured using the Uvitec Cambridge gel doc system and software (UVITEC Cambridge Ltd. Innovation Centre, Cambridge, Cambridge, UK) and the Kodak Gel Logic imaging system (Kodak).

### Seahorse Analysis

Oxygen consumption rate (OCR) and extracellular acidification rate (ECAR) under IgG or T-DXd treatment for 2 or 72 h were determined using a Seahorse XF96 Extracellular Flux Analyzer (Agilent Technologies). 96 h prior to the assay, 6,200 SKBR3 cells/well were seeded in XF96 plates. 72 or 2 h prior experiment, SKBR3 cells were treated either with 10 μg/mL IgG, Trastuzumab (Tz) or T-DXd. OCR and ECAR were monitored according to the manufacturer’s instructions. Briefly, the day of the experiment, the medium was replaced with Agilent Seahorse DMEM, pH 7.4, enriched with glucose (10 mM), glutamine (2 mM) and pyruvate (1 mM). A baseline recording followed by sequential injection of the ATP-synthase inhibitor oligomycin A (1.5 μM), the ATP synthesis uncoupler carbonyl cyanide-4-trifluoromethoxyphenylhydrazone (FCCP) (2 μM) and the Complex I and III inhibitor mix Rotenone/Antimycin A (0.5 μM) were recorded in 5 or 10 replicates. Three measurements of OCR and ECAR were taken for each condition and normalized to the IgG-treated controls.

### Reactive Oxygen Species Measurement

To determine the H_2_O_2_ production, 80 000 cells/well were seeded in a 24-well plate and incubated for 24 h in DMEM culture medium without phenol red supplemented with 2 μM H_2_DCFDA (ThermoFisher Scientific, D399) for 30 min at 37 °C. Afterward, the incubation medium was removed, and cells were incubated with DMEM medium without phenol red in the presence of IgG or T-DXd (10 μg/mL). Fluorescence (excitation, 485 nm; emission, 535 nm) was detected using a multilabel plate reader Victor3 (PerkinElmer, Turku, Finland) immediately (0 h) and after 2, 4, 6 and 20 h of treatment. Fluorescence values were subtracted of blank (cells without H_2_DCFDA probe) and normalized to live cell number. Calcein AM staining (100 nM, ThermoFisher Scientific, C3100MP) was used to determine live cell number in a twin parallel 24-well plate. Briefly, the dye was incubated for 30 min at 37 °C and DMEM medium without phenol red was replenished. Fluorescence was detected (excitation, 485 nm; emission, 535 nm) at the same time points of ROS measurement. Each experiment was performed in duplicate wells for each condition with at least 3 biological replicates. Treatment with *tert*-butylhydroperoxide (300 nM; Sigma-Aldrich, 458139-100 ML) was used as positive control of H_2_O_2_ production.

### Lysosome Purification

Lysosomes were isolated from approximately 3 × 10^7^ SKBR3 cells using the *Minute Lysosome Isolation Kit* (Invent Biotechnologies, Cat. No. LY-034), according to the manufacturer’s protocol with minor adjustments. All procedures were performed on ice or at 4 °C. Briefly, cells were harvested by centrifugation at 500*g* for 5 min, washed once with cold PBS, and resuspended in 500 μL of Buffer A supplemented with protease and phosphatase inhibitors (Complete Mini and PhosSTOP, Roche). After incubation on ice for 10 min, the suspension was vortexed vigorously for 20–30 s to disrupt the plasma membranes and frozen twice at −80 °C. Then, they were immediately transferred to a prechilled filter cartridge provided with the kit. The cartridge was capped, inverted a few times, and centrifuged at 16 000*g* for 30 s and transferred again in the filter. The flow-through was vortexed and centrifuged at 2000*g* for 3 min to remove nuclei and unbroken cells. The resulting supernatant was transferred to a clean microcentrifuge tube and centrifuged at 8000*g* for 15 min at 4 °C to pellet mitochondria and debris. The clarified supernatant (∼400 μL) was then centrifuged at 16 000*g* for 30 min at 4 °C to collect the lysosome-enriched fraction. The pellet was resuspended in 200 μL of cold Buffer A by repeated pipetting (60–100 times) and brief vortexing, followed by centrifugation at 2000*g* for 4 min. The resulting supernatant was mixed with Buffer B (2:1 v/v), incubated on ice for 30 min, and centrifuged at 11 000*g* for 10 min. The final pellet, corresponding to the isolated lysosomal fraction, was washed once with cold Buffer A and solubilized in 50–150 μL of EB buffer for subsequent protein analysis plus protease and phosphatase inhibitors. Lysosome enrichment and purity were verified by Western blotting using anti-LAMP1 and anti-MTFR1 antibodies in comparison with total cell lysates. For proteomic analysis, the final pellet was immediately frozen dried, and next digested with sequencing-grade trypsin, and subjected to LC-MS/MS analysis.

### Proteomic Analysis

Lysosome pellets were received as frozen, dry material in Eppendorf tubes and subsequently lysed in 50 μL of iST-LYSE buffer (PreOmics GmbH) for 10 min at 95 °C on an Eppendorf ThermoMixer (1000 rpm). After lysis, samples were sonicated (3 × 30 s cycles), and protein concentration was determined using a tryptophan fluorescence assay on a Tecan I-Control spectrophotometer. Protein isolation and digestion were performed using an automated Protein Aggregation Capture (PAC)-based protocol^45^ on a KingFisher Apex magnetic handling station (Thermo Fisher Scientific) in 96-well format. Briefly, a 1:1 mixture of Sera-Mag SpeedBead Carboxylate-Modified Magnetic Particles (Cytiva; cat. 45152105050250 and 65152105050250) was added to the lysates at a protein-to-bead ratio of 1:4 (w/w). Protein aggregation was induced by addition of 70% acetonitrile (ACN), followed by sequential washing steps with 100% ACN, 70% ethanol, and 100% isopropanol. On-bead digestion was carried out in 100 μL of 25 mM Tris–HCl (pH 8.0) using Lys-C (1:400 w/w, Wako) and trypsin (1:200 w/w, Promega) for 2.5 h at 37 °C. Reactions were stopped by adding 10 μL of 2% trifluoroacetic acid (TFA). Peptides were desalted using the in-StageTip (iST) method [Kulak et al., 2014], dried under vacuum centrifugation, and reconstituted in 2% ACN and 0.1% formic acid (FA).

### LC–MS/MS Analysis

Peptide samples were analyzed using an UltiMate 3000 RSLCnano system coupled to a Q Exactive Plus mass spectrometer (Thermo Fisher Scientific). Chromatographic separation was performed on an EASY-Spray PepMap RSLC C18 column (25 cm × 75 μm i.d., 2 μm, 100 Å) at 250 nL/min using a nonlinear gradient from 2% to 45% buffer B (80% ACN, 20% H_2_O, 5% DMSO, 0.1% FA) over 50 min. Buffer A consisted of 0.1% FA in water. The instrument operated in positive polarity and data-independent acquisition (DIA) mode. Full MS scans (*m*/*z* 375–1500) were acquired in the Orbitrap at 70,000 resolution (AGC target 3 × 10^6^). Precursor ions were isolated with 34 *m*/*z* windows (19 loops) and fragmented using higher-energy collisional dissociation (HCD) at 27% normalized collision energy. MS2 spectra were acquired at 35,000 resolution (AGC target 3 × 10^6^).

### Protein Identification and Quantification

Raw DIA data were processed in Spectronaut v18 (Biognosys AG) using DirectDIA mode with default library-free settings. Spectral searches were performed against the UniProt *Homo sapiens* reviewed (canonical) FASTA database. Carbamidomethylation of cysteines was set as a fixed modification, while N-terminal acetylation, methionine oxidation, and asparagine/glutamine deamidation were defined as variable modifications. Protein inference included Gene Ontology (GO) annotations derived within Spectronaut. Identifications were filtered at a 1% false discovery rate (FDR) at both the protein and precursor levels. Quantitative intensities were log_2_-transformed and normalized using Spectronaut’s local normalization algorithm to correct for intrarun variability. Only protein groups consistently detected in all three biological replicates per condition were retained for downstream analyses. Normalized data matrices were exported for statistical evaluation in Perseus v1.6.15.0 and for visualization in R v4.4.3 using the *ggplot2* and *ComplexHeatmap* packages. The Protein Quant Pivot Report generated by Spectronaut was used for statistical analysis in Perseus.

### Bioinformatic and Statistical Analysis

Missing values were handled using the Run-wise Imputing strategy implemented in Spectronaut, optimized for library-free DIA workflows. Differential protein expression between T-DXd-treated and IgG-treated lysosome samples was assessed using the LIMMA package in R after log_2_ transformation of normalized intensities. Global protein expression patterns were visualized by hierarchical clustering (Euclidean distance, average linkage) of z-scored log_2_ intensities across proteins and samples. Heatmaps display z-scored abundances, with rows representing proteins and columns representing biological replicates, highlighting treatment-specific expression signatures.

### Functional Enrichment and Pathway Analysis

Functional enrichment of differentially expressed proteins was performed using WebGestalt (WEB-based Gene SeT AnaLysis Toolkit; www.webgestalt.org). Gene Set Enrichment Analysis (GSEA) was carried out using the ranked list of proteins based on log_2_ fold change between TxDXd and IgG treatments. Analyses were conducted against the *Homo sapiens* reference set using Gene Ontology (Biological Process, Molecular Function, and Cellular Component) and Reactome pathway databases. The minimum and maximum gene set sizes were set to 15 and 2000, respectively. Significantly enriched categories were identified using an FDR < 0.05 (Benjamini–Hochberg correction). Normalized enrichment scores (NES) and FDR values were used to rank pathways. The top enriched GO terms and Reactome pathways were visualized in R using *ggplot2* and *enrichplot*, and summarized into nonredundant clusters based on semantic similarity of GO terms.

### Cytokine Measurement

The median values of the concentrations of the cytokines examined were compared across 4 different cell lines (SKBR3, BT474, MDA-MB-231, and MDA-MB-361), comparing in the presence of T-DXd or IgG. For the lines treated with the T-DXd, we diversified the drug exposure: 72 h and 10 days. The control used was composed of only the medium used for cell cultures with the addition of T-DXd (DMEM 10% serum + T-DXd and DMEM 20% serum + T-DXxd) or IgG (DMEM 10% serum + IgG and DMEM 20% serum + IgG). The cellular supernatant was collected in 15 mL Falcon tubes, aliquoted into 2.5 mL criovials, and stored at −80 °C. The samples were diluted twice, according to protocol with the sample diluent, and centrifuged for 15 min at 1000*g*, then inserted into the Ella Simple Plex cartridge (ProteinSimple, San Jose, CA, USA). The cartridge was then placed inside the reactor and left to operate for 90 min at room temperature. The concentrations were expressed in pg/mL. All samples were centrally analyzed at the Translational Research Laboratory of the ARCO Foundation at the S. Croce and Carle Hospital in Cuneo, Italy, and evaluated in triplicate.

### Cytokine Statistical Analysis

The statistical analyses were conducted using GraphPad Prism 5 (GraphPad Software, Boston, Massachusetts, USA) and SPSS V.24 (IBM SPSS Statistics for Windows, Version 24.0. Armonk, NY, USA). The concentrations have been converted to scale 10 logarithm to avoid zeros. The ratio was calculated by dividing the T-DXd concentrations by the IgG concentrations. Statistical significance for transcript analyses was determined using the unpaired *t* test with Welch’s correction. Data are expressed as mean ± SEM, and the scale used is logarithmic base 10. In all tests, a *p*-value of 0.05 or lower was considered significant. The Benjamini–Hochberg (B–H) procedure was applied to reduce the false positive rate to 25% (x).^46^ If not clear, the *p*-value was considered not significant (NS) for this percentage.

## Results

### T-DXd Temporally Modulates HER2 Phosphorylation, Downstream Signaling and Lysosomal Biogenesis

Since HER2/HER2 trafficking and signaling are key determinants of ADC efficacy, we investigated the effects of T-DXd on HER2 phosphorylation and downstream signaling in SKBR3 (Figure 1), BT474, and MDA-MB-361 HER2+ BCa cell lines (Figure S1A,B). Cells were treated with 10 μg/mL T-DXd for 2 and 72 h and with IgG as control, and the signaling response was analyzed by immunoblotting, focusing on HER2 phosphorylation at Y1248, total HER2 levels, and the downstream HER2-dependent pathways, including pAKT (Ser473) and pERK1/2. Our results revealed a biphasic, time-dependent modulation of HER2 phosphorylation and downstream signaling cascades (Figure 1A,B). Notably, at 72 h, phosphorylation of the residual HER2 pool at Y1248 and detectable pERK1/2 signals were maintained. This suggests relative preservation of HER2–ERK signaling activity despite receptor downregulation, potentially reflecting signaling from residual membrane-associated and/or intracellular HER2 pools. Interestingly, this effect appeared more evident in BT474 and MDA-MB-361 cells (Figure S1A,B), suggesting cell line–dependent differences in signaling persistence following T-DXd treatment. This result is consistent with previous work, where Trastuzumab treatment led to a shift in signaling dynamics, favoring pERK activation while suppressing pAKT.^47^ As lysosomes play a crucial role in ADC trafficking and payload release, we next investigated whether T-DXd modulates lysosomal and autophagic responses. We analyzed the expression of TFEB, a master regulator of lysosomal biogenesis and autophagy, LC3-I/II, markers of autophagosome formation, and SQSTM1/p62, an autophagic cargo adaptor involved in protein degradation pathways.^48^ At 2 h, we observed a transient increase in TFEB, along with reductions in LC3I/II and LAMP1, while SQSTM1/p62 remained unchanged, suggesting an early lysosomal-autophagic remodeling response induced by T-DXd treatment. By 72 h, LC3I/II and LAMP1 levels rose above IgG-treated controls, while TFEB returned to baseline, indicating dynamic temporal changes in lysosomal-autophagic markers during T-DXd exposure (Figure 1C). To better distinguish trastuzumab-associated signaling effects from ADC-specific responses, we also analyzed HER2 signaling and lysosomal-autophagic markers in SKBR3 cells treated with trastuzumab alone (Figure S2). Trastuzumab treatment induced a trend of reduction in pAKT levels together with persistent pERK1/2 and HER2 Y1248 phosphorylation relative to total HER2, consistent with signaling rewiring downstream of HER2 engagement (Figure S2A,C)). In contrast, markers associated with DNA damage and late stress responses, including γH2AX, LaminB1, and lysosomal-autophagic proteins, showed no major changes, as opposed to after T-DXd treatment (Figure S2B,C). These findings suggest that trastuzumab contributes to early HER2-dependent signaling and metabolic remodeling, whereas the DXd payload additionally drives a stress-associated organelle phenotype. As deruxtecan induces apoptosis in treated cells, we assumed that this death process is also elicited by treatment with T-DXd. Flow cytometry analysis revealed that T-DXd efficiently induces apoptosis in SKBR3 and MDA-MB-361 cells, showing a significantly higher early and late apoptotic response compared to trastuzumab-treated and IgG controls (Figure 1E and Figure S1B,C). In contrast, BT474 cells treated with T-DXd exhibited a lower early apoptotic response when compared to the trastuzumab treatment, yet remaining more responsive respect to the IgG treatment, suggesting a differential sensitivity to T-DXd-induced apoptosis across HER2+ BCa models (Figure 1E and Figure S1B,C).

**Figure 1:**
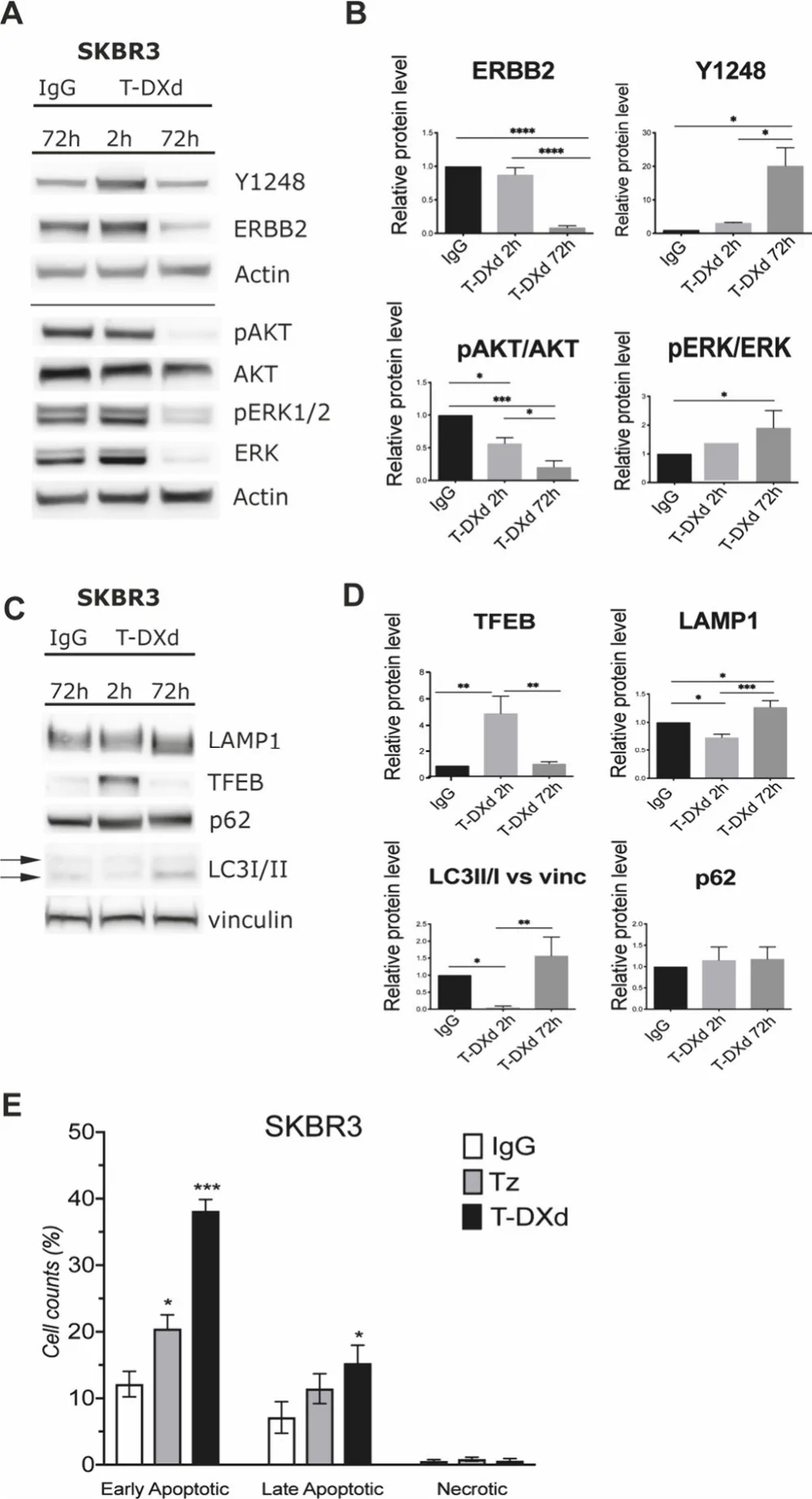
Time-dependent modulation of HER2 signaling, lysosomal response, and apoptosis upon T-DXd treatment in HER2+ breast cancer cells. (A) Immunoblot analysis of HER2 phosphorylation at Y1248, total HER2 levels, and downstream signaling pathways (pAKT S473 and pERK1/2) in SKBR3 cells treated with 10 μg/mL T-DXd for 2 and 72 h, compared to IgG control. Actin was used as a loading control. (B) Quantification of Western blot in A. Phosphorylated proteins were first normalized to their corresponding total protein levels (pHER2 Y1248/total HER2, pAKT S473/total AKT, and pERK1/2/total ERK1/2) and then expressed relative to the corresponding IgG-treated control. All other protein levels were normalized to actin and expressed as relative optical density compared with IgG-treated controls. Data are presented as mean ± SEM from three independent experiments (*N* = 3). Statistical significance was determined using one-way ANOVA. (C) Immunoblot analysis of TFEB, LAMP1, LC3I/II, and p62 in response to T-DXd treatment. Actin was used as a loading control. (D) Quantification of Western blot in C. Each bar graph represents the mean relative optical density quantification relative to IgG-treated control. LC3I/II was quantified as ratio between LC3II/I versus vinculin. Error bar + SEM. Significance was determined using one-way ANOVA from *N* = 3 independent experiments. (E) Flow cytometry analysis of apoptosis in SKBR3 cells treated with T-DXd, trastuzumab, or IgG treated controls for 72 h. The percentage of early and late apoptotic cells, and necrotic cells after 72 h of exposure to T-DXd, trastuzumab or IgG is shown. Annexin-V-APC^+^/Sytox Blue^–^, Annexin-V-APC^+^/Sytox Blue^+^, and Annexin-V-APC^–^/Sytox Blue^+^ cells were identified as early apoptotic, late apoptotic and necrotic cells, respectively. Mean values and SD (indicated as vertical bars) (*n* = 3) are shown. Statistical significance: *P* < 0.01 (**), *P* < 0.001 (***), *P* < 0.0001 (****).

### T-DXd Induces Early Metabolic Activation and Late Mitochondrial Dysfunction Independent of ROS Production

Given the early downregulation of pAKT observed as early as 2 h post-treatment, alongside sustained phosphorylation of HER2 and detectable pERK1/2 up to 72 h, we examined whether these signaling dynamics influenced mitochondrial bioenergetics.^49^ Since AKT signaling plays a central role in regulating mitochondrial function, glucose metabolism, and cellular energy homeostasis,^50^ we investigated whether these signaling dynamics were associated with alterations in mitochondrial bioenergetics following T-DXd treatment. To assess mitochondrial function, we measured oxygen consumption rate (OCR) and extracellular acidification rate (ECAR) using a Seahorse Extracellular Flux Analyzer in SKBR3 cells treated with T-DXd and Trastuzumab (Tz) for 2 and 72 h (Figure 2A–D). At 2 h post-treatment, T-DXd-treated cells exhibited a significant increase in maximal mitochondrial respiration and nonmitochondrial respiration, whereas basal respiration and ATP-linked respiration remained unchanged (Figure 2A), indicating an early metabolic response associated with T-DXd exposure (Figure 2A). ECAR was also elevated, indicating a concomitant increase in glycolytic activity (Figure 2B). In contrast, trastuzumab-treated cells showed metabolic profiles comparable to IgG controls at this early time point. This early metabolic activation correlates with the transient increase in HER2 phosphorylation observed at 2 h, suggesting that enhanced HER2 signaling may drive a transient adaptive metabolic response. The observed increase in both OXPHOS and glycolysis may reflect a short-term survival strategy, allowing cells to rapidly generate energy in response to treatment-induced stress. At 72 h, both trastuzumab and T-DXd treatments were associated with a marked reduction in OCR and ECAR parameters, consistent with progressive metabolic impairment. However, the metabolic suppression induced by T-DXd was more pronounced, indicating a more severe energetic collapse likely associated with additional ADC-related intracellular stress mechanisms beyond HER2 blockade alone.

**Figure 2:**
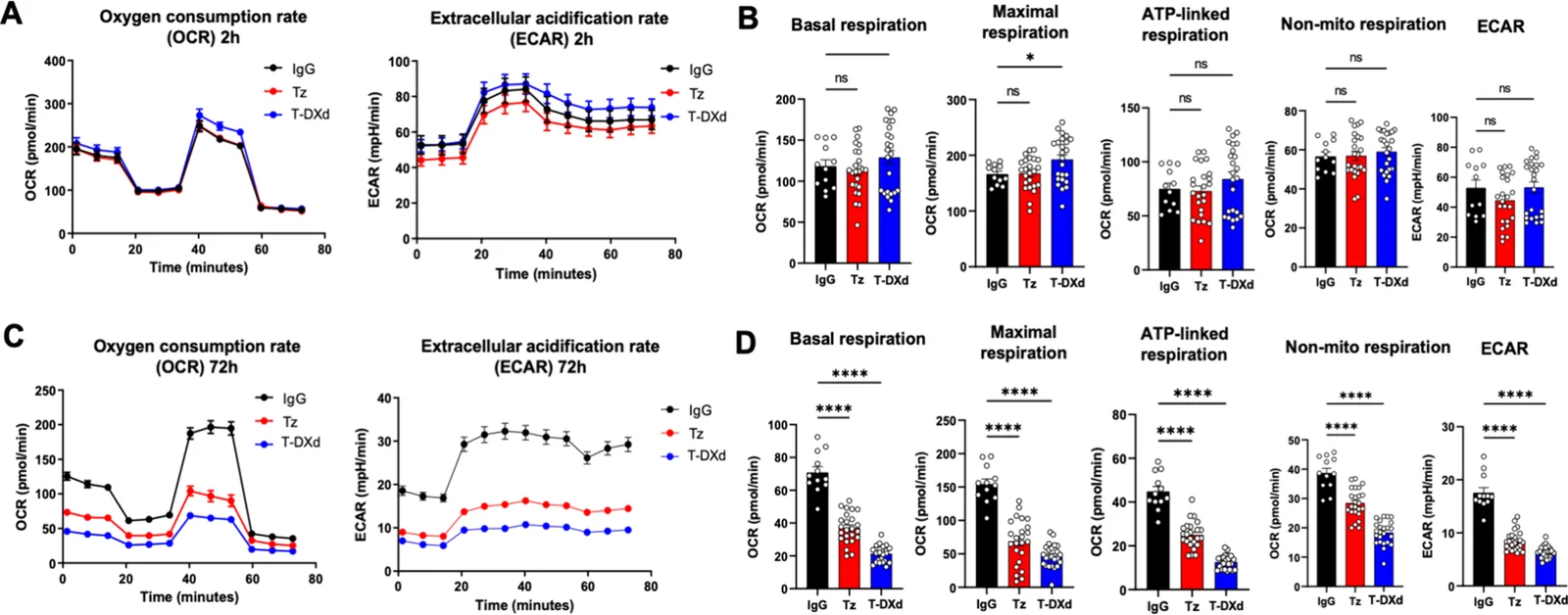
Time-dependent metabolic adaptations induced by T-DXd treatment in SKBR3 cells. (A,B) Left, Average oxygen consumption rate (OCR) and extracellular acidification rate (ECAR) kinetics showing the response of SKBR3 cells to 10 μg/mL T-DXd and Tz for 2 h compared to IgG-treated cells. Mean ± SEM. Right, Quantification of basal, maximal, ATP-coupled respiration, and nonmitochondrial oxygen consumption (OCR) and ECAR acidification rate (ECAR). 2 h post-treatment, OCR measurements show a significant increase in maximal respiration and nonmitochondrial respiration, accompanied by an elevation in ECAR. *N* = 3 independent experiments, 30 replicates/condition. (C,D) Left, Average OCR and ECAR kinetics showing the response of SKBR3 cells to 10 μg/mL T-DXd and Tz for 72 h. Mean ± SEM. Right, Quantification of basal, maximal, ATP-coupled respiration, and nonmitochondrial oxygen consumption (OCR) and ECAR acidification rate (ECAR). 72 h post-treatment, oxygen consumption rate (OCR) measurements show a significant decrease in basal, ATP-linked, maximal respiration and nonmitochondrial respiration, accompanied by a reduction in extracellular acidification rate (ECAR). *N* = 3 independent experiments, 30 replicates/condition. Data normalization was performed over IgG controls. Statistical significance: *P* < 0.01 (**), *P* < 0.001 (***), *P* < 0.0001 (***).

To determine whether this metabolic shift was associated with oxidative stress, we measured H_2_O_2_ production and the abundance of 4-hydroxynonenal (4-HNE), the end product of lipid peroxidation. Interestingly, T-DXd did not induce a significant increase in either parameter, despite the metabolic activation observed at 2 h, indicating that the early metabolic enhancement occurs independently of oxidative stress (Figure S3A,C). At later stages, a metabolic remodeling together with a modest oxidative stress responses may contribute to cellular adaptation, as suggested by the significant increase in the antioxidant SOD2 protein levels (Figure S3B). In line with these results, at 72 h post-treatment, both OCR and ECAR were significantly suppressed, shifting the energetic profile of SKBR3 cells toward a preapoptotic state (Figure 2C,D). Since metabolic shifts are often accompanied by mitochondrial remodeling, we next examined mitochondrial network organization by confocal microscopy following T-DXd treatment. Morphometric analysis revealed an increase in total mitochondrial area, mitochondrial branch length, and branch junctions, consistent with extensive mitochondrial network reorganization (Figure 3A–D). Consistently, EM morphometric analysis showed elongated and highly interconnected mitochondria displaying altered cristae organization (Figure 3E,F, asterisk).

**Figure 3:**
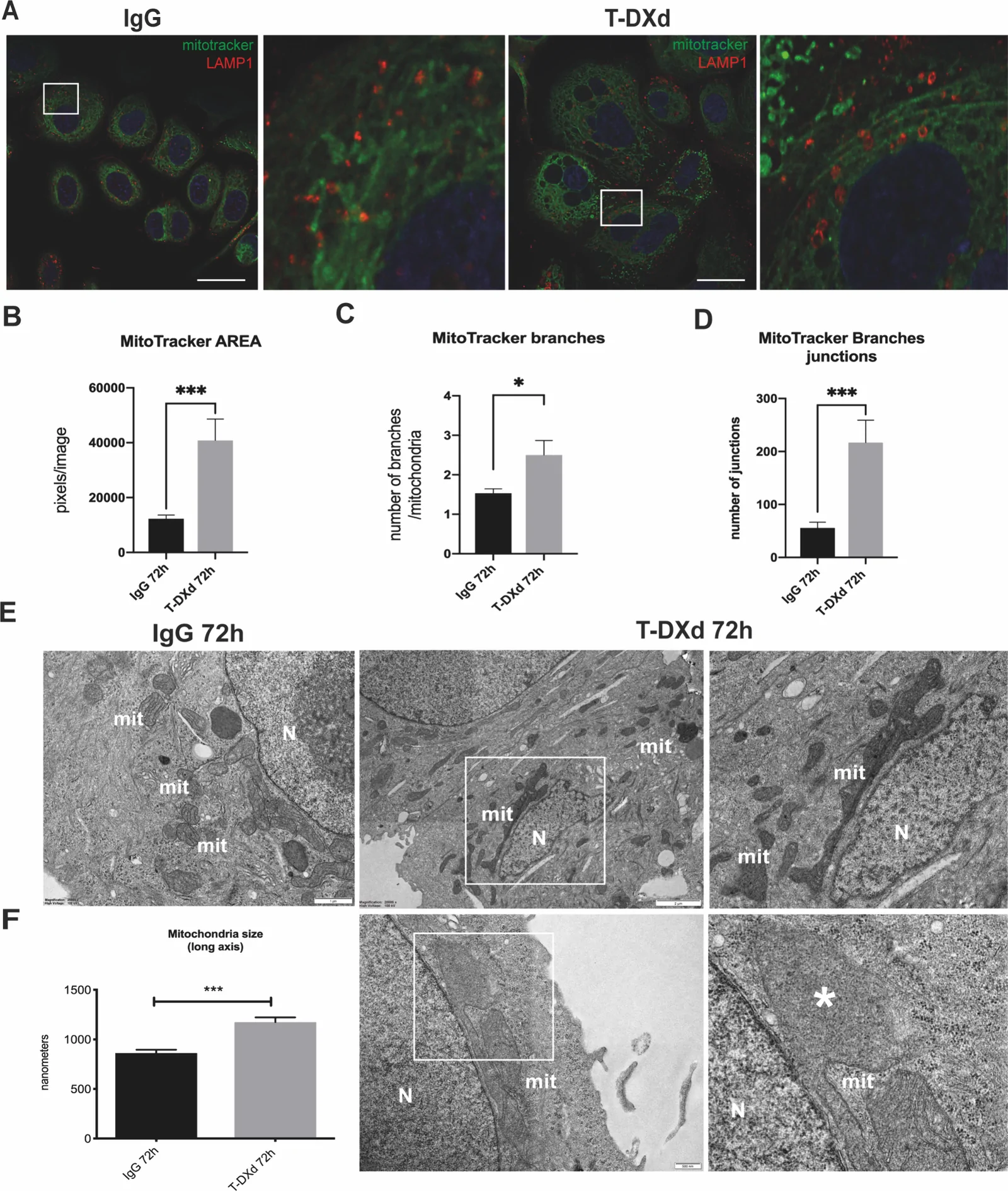
Mitochondrial network dynamics upon T-DXd treatment in SKBR3 cells. (A) Confocal microscopy images of SKBR3 cells treated with IgG or T-DXd for 72 h, stained with MitoTracker Green and LAMP1 antibody. T-DXd-treated cells exhibit mitochondrial disorganization and an extended, fused mitochondrial network. Scale bars: 10 μm. (B) Quantification of MitoTracker morphology in IgG- and T-DXd-treated cells at 72 h. Morphometric analysis of mitochondrial area (B), branches (C), and branch junctions (D) was performed using the Mitochondria Analyzer ImageJ plugin. Data are presented as mean ± SEM from three independent experiments, with 50 cells analyzed per condition (unpaired *t* test). (E) Representative electron microscopy (EM) images of SKBR3 cells treated with IgG or T-DXd for 72 h. In T-DXd-treated cells, mitochondria appear fused and display cristae alterations and swelling. N: nucleus; mit: mitochondria. The asterisk indicates a mitochondrial region with poorly preserved or no longer discernible cristae (F). Quantification of mitochondrial long axis length by EM morphometry. Mitochondria were measured in 30 whole cells per experimental condition (see material and methods) and plotted as histograms (mean ± SEM). *N* = 3 independent experiments. Statistical significance was determined using the Mann–Whitney test.

### Spatiotemporal Emergence of Lysosome–Mitochondria Contacts and Lysosome–Mitochondria–Nucleus Triads during T-DXd Internalization

To evaluate HER2-associated trafficking dynamics in response to T-DXd, we performed real-time live-cell imaging using the IncucyteSX5 system for over 72 h. SKBR3 cells were treated with T-DXd and stained with an anti-HER2 antibody conjugated to CoraLitePlus488. Raw quantification of internalization (HER2 Count per image) revealed an increase in HER2-associated fluorescent puncta in T-DXd-treated cells compared with IgG-treated controls (Figure 4A,B). Since this measurement can be influenced by changes in cell number, cell confluence was quantified from the phase-contrast channel (Figure 4C), showing that T-DXd-treated cells increased in confluence up to approximately 48 h and subsequently declined, whereas IgG-treated controls continued to grow over time. HER2 object count was therefore normalized to cell confluence area at each time point (Figure 4D). The confluence-normalized analysis confirmed a marked increase in HER2-associated puncta following T-DXd exposure, with elevated signal up to approximately 48–60 h and a decline at 72 h. This late decrease was consistent with the reduction in cell confluence observed at the same time point, suggesting loss of viable/adherent cells during prolonged T-DXd exposure. To further assess intracellular trafficking, immunofluorescence analysis was conducted at 2, 24, 48, and 72 h post-treatment. Co-localization between T-DXd and LAMP1 increased over time, particularly in perinuclear regions, suggesting progressive lysosomal accumulation. At 48 and 72 h, nuclear alterations, such as fragmentation and envelope deformation, were observed, consistent with DNA damage following lysosomal processing (Figure 4E,F). Consistently, flow cytometry analysis using LysoTracker confirmed the increase in lysosomal-associated fluorescence between 48 and 72 h of T-DXd treatment, supporting the progressive expansion and/or acidotropic accumulation of the lysosomal compartment at late time points (Figure 4G).

**Figure 4:**
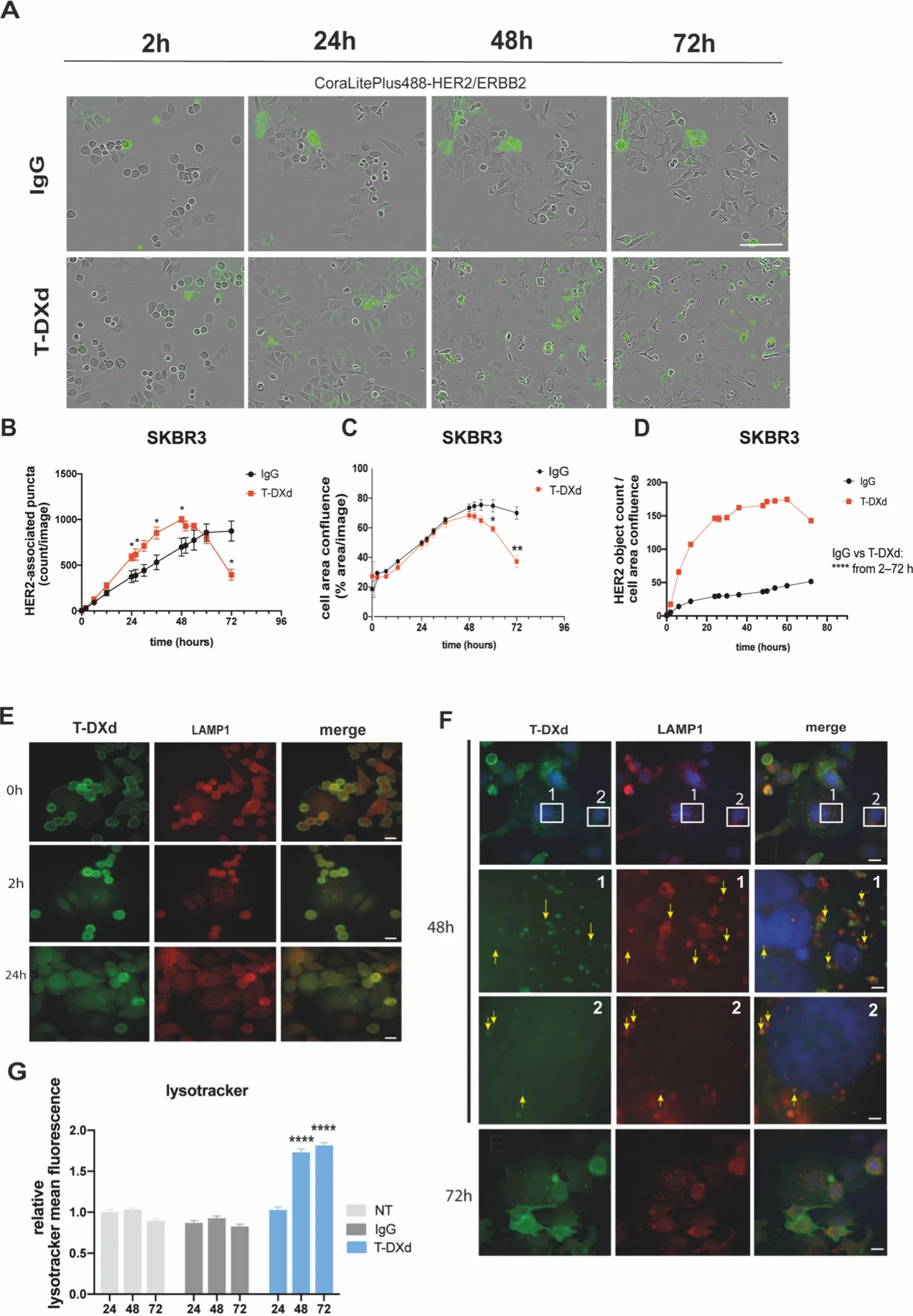
T-DXd internalization and subcellular localization. (A) Real-time live-cell imaging of SKBR3 cells treated with T-DXd over 72 h using the IncucyteSX5 system. Scale bars: 200 μm. (B) Cell proliferation is shown as % of cell confluence. (C) HER2 internalization was monitored with an anti-HER2 antibody directly conjugated to CoraLite488 and quantified as HER2-associated fluorescent puncta per image. (D) Confluence-normalized quantification of HER2-associated fluorescent puncta. HER2 CoraLite Count per image was normalized to the corresponding phase-contrast-derived cell confluence area at each time point and then expressed relative to the initial time point. *N* = 3 experiments, 3 replicates each condition, 4 images/well. Statistical significance was determined by two-way ANOVA followed by Sidak’s multiple comparisons test comparing IgG and T-DXd at each time point. *P* < 0.05 (*), *P* < 0.0001 (****). (E, F) Immunofluorescence microscopy images of T-DXd-treated SKBR3 cells at 2, 24, 48, and 72 h post-treatment. Cells were stained with an anti-LAMP1 antibody (red) to assess lysosomal localization and antihuman cy2 (green) to track T-DXd. Co-localization of T-DXd with LAMP1 (yellow arrows) was observed in the cytoplasm and near the nucleus, along with nuclear fragmentation. Boxes labeled 1 and 2 indicate regions shown at higher magnification in the corresponding enlarged panels below. DAPI staining is shown only in selected late-stage merged images (48–72 h) to highlight treatment-associated nuclear alterations. Scale bars: 20 μm. (G) Flow cytometry analysis of LysoTracker fluorescence intensity in SKBR3 cells treated with IgG or T-DXd for 24, 48, and 72 h, compared to untreated control cells (NT). Data are presented as mean fluorescence intensity (MFI). Results represent the mean ± SD of three independent experiments (*n* = 3), each performed in technical triplicate. Statistical analysis was performed using two-way ANOVA, with the following significance levels: *P* < 0.05 (*), *P* < 0.01 (**), *P* < 0.001 (***), *P* < 0.0001 (****). Only most important pairwise comparisons are shown. Full statistical analysis and results are included in Supplementary Table 1.

In addition, ultrastructural quantitative morphometric analysis of lysosomes, identified using 5 nm BSA gold nanoparticles as endocytic tracer, confirmed a significant increase in average lysosomal size over time, indicating lysosomal expansion (Figure S4A,B). Notably, starting at 24 h, we observed the progressive formation of lysosome–mitochondria (Figure S4D) and lysosome–mitochondria–nucleus nanoscale contacts (Figure S4E), particularly near distorted nuclei.^46,51^ For quantitative analysis, contacts were defined as membrane appositions with an interorganelle distance ≤30 nm, measured on TEM micrographs.^52^ This architecture was further validated using nanogold-labeled LAMP1, which confirmed lysosomal contacts with mitochondria and the nuclear envelope (Figure S4F).

Consistent with the observations obtained using BSA–gold and LAMP1 labeling, direct nanoscale tracking using horseradish peroxidase (HRP)-conjugated trastuzumab and T-DXd confirmed that lysosome-associated HER2-targeting agents establish close contacts with mitochondria and the nuclear envelope (Figure 5A,B). At 24 h, both trastuzumab- and T-DXd-positive lysosomes frequently formed contact sites with mitochondria and displayed budding profiles, consistent with active lysosomal remodeling (Figure 5E). However, quantitative ultrastructural analysis revealed that T-DXd treatment induced a more pronounced and progressive increase in lysosomal number and diameter compared with trastuzumab alone, reaching maximal enlargement at 48 h and remaining elevated at later time points (Figure 5C,D). In parallel, the frequency of lysosome–mitochondria–nucleus triads, although representing relatively rare ultrastructural events, was markedly higher in T-DXd-treated cells, particularly at 48 h, whereas trastuzumab alone induced only modest increases in these multiorganelle interactions (Figure 5F). By 48–72 h, T-DXd-positive lysosomes frequently contained electron-dense intraluminal material and were closely associated with altered mitochondria and the nuclear envelope, consistent with progressive lysosome-associated organelle remodeling during prolonged ADC exposure.

**Figure 5:**
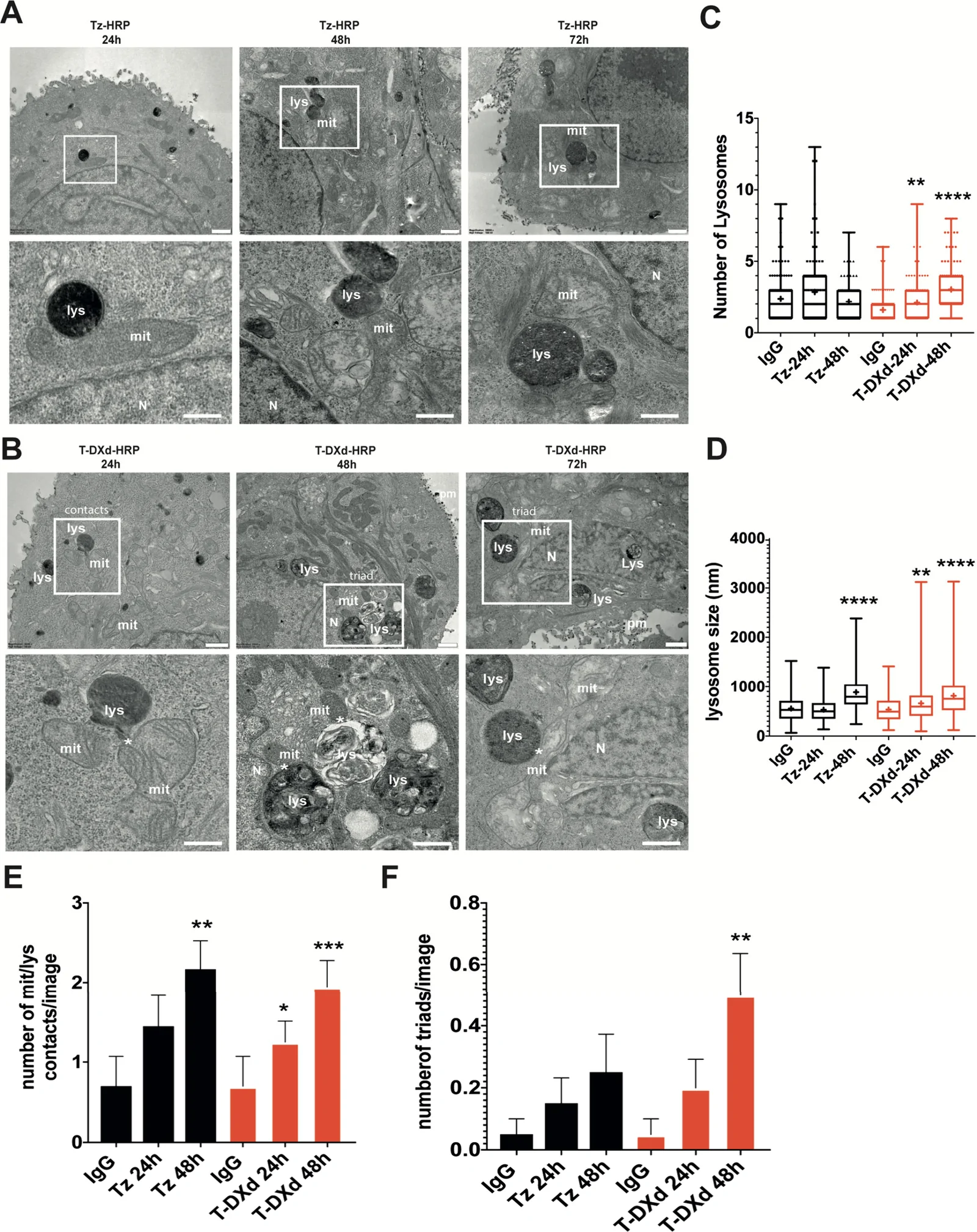
Ultrastructural analysis of lysosomal remodeling and organelle interactions induced by trastuzumab and T-DXd in HER2+ breast cancer cells. (A) Representative TEM micrographs of SKBR3 cells treated with HRP-conjugated trastuzumab (Tz) for 24, 48 and 72 h, followed by DAB development, showing HRP-positive lysosomal structures (lys) and their interactions with mitochondria (white boxes). (B) Representative TEM micrographs of SKBR3 cells treated with HRP-conjugated T-DXd for 24, 48, and 72 h. T-DXd-positive lysosomes appeared enlarged, frequently associated with mitochondria and the nuclear envelope, and contained electron-dense intraluminal material at later time points. (C) Quantification of lysosome number per image in IgG-, Tz-, and T-DXd-treated cells at 24 and 48 h. Data are presented as box plots with individual data points. Trastuzumab induced a modest transient increase in lysosome number, whereas T-DXd treatment resulted in a more pronounced increase at 24 and 48 h. (D) Quantification of lysosomal size in IgG-, Tz-, and T-DXd-treated cells. Tz induced a significant increase in lysosomal size at 48 h (****p < 0.0001), whereas T-DXd treatment induced a pronounced enlargement at both 24 and 48 h (**p < 0.01, ****p < 0.0001). (E) Quantification of lysosome–mitochondria contact sites per image (*n* = 30 images). Trastuzumab significantly increased the frequency of lysosome–mitochondria contacts at 48 h (**p < 0.01), while T-DXd induced a progressive increase at both 24 h (*p < 0.05) and 48 h (***p < 0.001). (F) Quantification of lysosome–mitochondria–nucleus triads per image (*n* = 30 images). Although triads represented relatively infrequent ultrastructural events, their frequency was significantly increased following T-DXd treatment at 48 h (**p < 0.01), whereas trastuzumab alone induced only modest changes. Statistical analyses were performed using Kruskal–Wallis test followed by Dunn’s multiple comparisons test. Abbreviations: Lys, lysosome; Mit, mitochondrion; N, nucleus; NE, nuclear envelope; PM, plasma membrane. Asterisks (*) indicate lysosome–mitochondria contact sites.

### T-DXd Induces DNA Damage and Nuclear Envelope Remodeling

Following T-DXd internalization and intracellular trafficking, we investigated its effects on nuclear integrity, focusing on DNA damage and nuclear envelope alterations. Western blot analysis of SKBR3 cells treated with T-DXd for 2 and 72 h revealed a marked upregulation of γH2AX protein levels at 72 h, indicative of DNA double-strand breaks (DSBs), while no significant change was observed at 2 h (Figure 6A). Quantification of γH2AX levels confirmed a significant increase at 72 h compared to IgG-treated controls (Figure 6B). Confocal microscopy at 72 h demonstrated the formation of γH2AX foci within the nuclei of T-DXd-treated cells, further confirming the presence of DSBs (Figure 6C). Quantification of these foci showed a significant increase in γH2AX foci per nucleus in T-DXd-treated cells compared to controls (Figure 6D). Next, we examined the nuclear lamina by analyzing LaminB1 expression levels and localization. Western blot analysis revealed a significant increase in LaminB1 expression at 72 h post-T-DXd treatment (Figure 6E). Consistently, IF analysis at 72 h showed its expected nuclear localization but also a redistribution, with a fraction of LaminB1 showing increased cytosolic presence and partial plasma membrane localization (Figure 6G). Furthermore, IF staining demonstrated nuclear enlargement, with a quantifiable increase in nuclear area (Figure 6F). As the upregulation of Lamin B1 observed at 72 h post-T-DXd treatment may represent a cellular attempt to stabilize the nuclear envelope in response to stress induced by the payload, we performed ultrastructural analysis by electron microscopy (EM) at 72 h. EM revealed nuclear abnormalities, including multilayered nuclear pores, membrane invaginations, and tube-like nuclear structures (Figure 6I). Additionally, fragmented nuclei were observed in T-DXd-treated cells, further confirmed by DAPI staining in IF.

**Figure 6:**
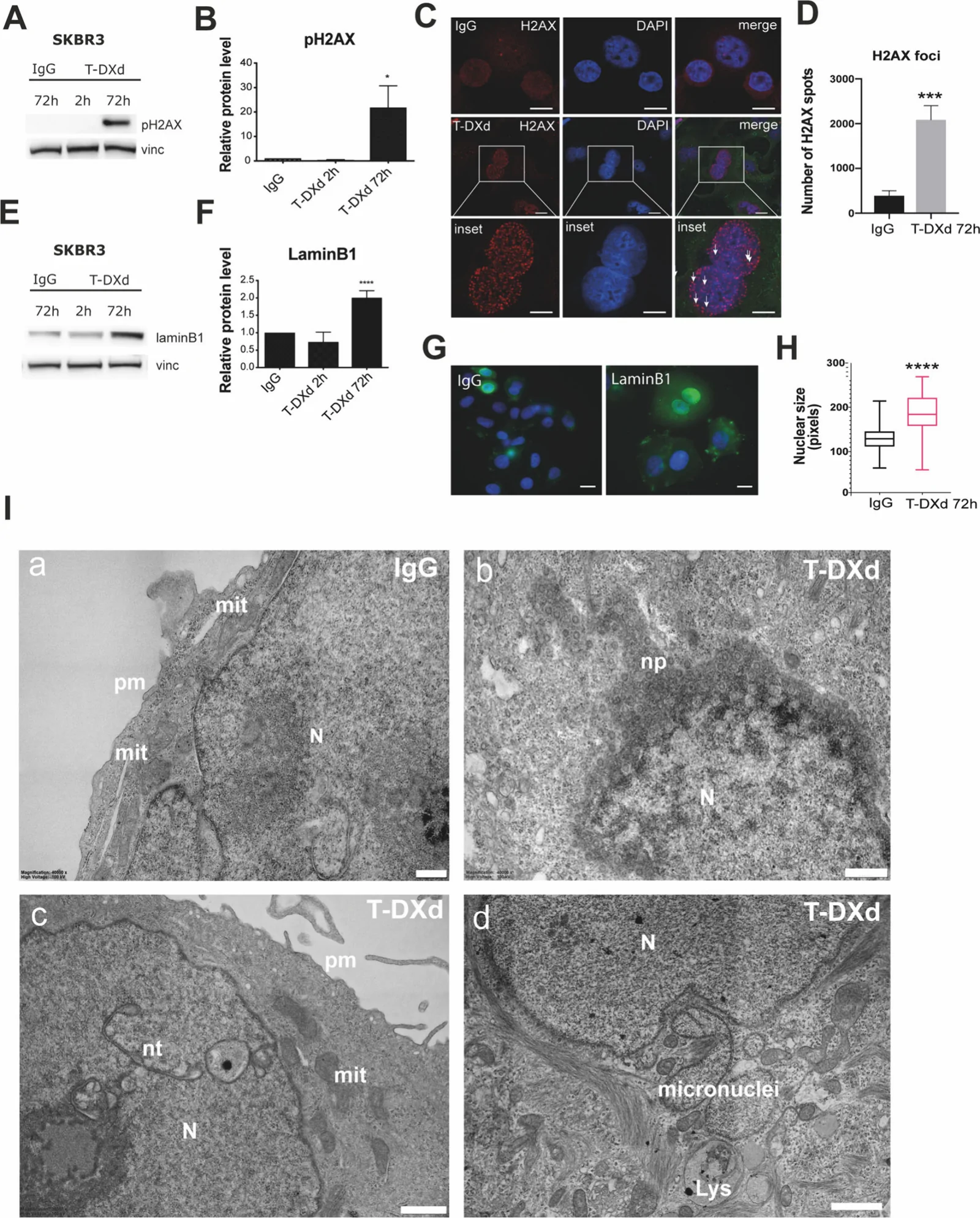
Assessment of nuclear damage and nuclear envelope alterations induced by T-DXd treatment. (A) Western blot analysis of γH2AX protein levels in SKBR3 cells treated with T-DXd at 2 and 72 h. Actin was used as a loading control. (B). Quantification of Western blot in A. Each bar graph represents the mean relative optical density quantification of γH2AX relative to IgG-treated control. Error bar + SEM. Significance was determined using one-way ANOVA from *N* = 3 independent experiments. (C) Confocal analysis of γH2AX foci formation in SKBR3 cells treated with IgG and T-DXd for 72 h. γH2AX foci, indicative of DNA double-strand breaks (DSBs), were detected using anti- γH2AX antibody (red). DAPI (blue) was used to detect nuclei. (D) Histogram showing quantification of γH2AX foci per nucleus. *N* = 3 independent experiments. Scale bars: 20 μm. (E) Immunoblot analysis of Lamin1B showing increased expression upon 72 h od T-DXd treatment (F) Quantification of LaminB1 expression from E. Each bar graph represents the mean relative optical density quantification of LaminB1 normalized to IgG-treated controls. Significance was determined using one-way ANOVA from *N* = 3 independent experiments. (G) Cells were stained with an anti-LaminB1 antibody (green) to assess nuclear envelope structure. Redistribution of LaminB1 was observed, with increased cytosolic presence and partial plasma membrane association. DAPI (blue) was used to detect nuclei. Scale bars: 20 μm. (H) Box plot (line at mean) showing nuclear size quantification by DAPI staining in IgG versus T-DXd treated cells for 72 h. *N* = 3 independent experiments. Statistical significance was determined using an unpaired *t* test. (I) Ultrastructural analysis of nuclear morphology in IgG and T-DXd-treated cells at 72 h. Representative EM images reveal several nuclear envelope abnormalities in T-DXd treated cells compared to IgG (a), including nuclear pore (np) multilayering (b), tube-like nuclear structures (nt) (c). Micronuclei are also observed (d). Uneven image brightness is due to MIA (Multiple Image Alignment) acquisition mode. *N* = nucleus, mit= mitochondrion, pm: plasma membrane. Scale bars: 2 μm (b), 1 μm (c), 500 nm (a,d).

Flow cytometry analysis revealed a significant increase in apoptotic cells at 72 h, indicating that nuclear alterations are closely associated with apoptotic progression (Figure 1E). These findings suggest that T-DXd induces nuclear morphological alterations, likely driven by persistent DNA damage and nuclear envelope remodeling. The observed changes suggest a broad nuclear stress response, which may contribute to the overall cytotoxic effects of T-DXd in HER2+ BCa cells.

### T-DXd Induces Changes in Protein Composition of Lysosomes

To characterize changes in the protein composition of the lysosomes induced after 48 h of treatment with T-DXd, we purified a subcellular fraction enriched in lysosomes and processed it for proteome analysis. We identified 4772 proteins and among them 380 proteins belonged to the LYS GS, GO:0005764 gene set, representing 46.2% of the total protein included in this gene set. Principal component analysis showed a clear separation of control samples from T-DXd treated samples but none of the identified proteins, except those T-DXd-related added to treated samples (IgG chains), reached a statistically significant fold change ratio ((|log 2FC| ≥ 2.5); p_thr = 1) (data not shown). However, when we performed gene set enrichment analysis (GSEA) we identified Cell cycle progression E2F targets, G2/M checkpoint, tumor necrosis factor (TNF-α) signaling via NF-kB, and DNA repair, PI3K signaling via AKT and KRAS downregulated genes as significantly modulated transcriptional pathways (FDR ≤ 0.05) (Figure 7A). Furthermore, GSEA performed to reveal biological processes, molecular function, and cellular component modulated by T-DXd, indicated an overall reduction of proteins related to biosynthetic and metabolic process and increase of proteins related to nuclear components and DNA repair mechanisms (Figure 7B–D). To reveal the most deregulated proteins, independently from statistical significance, we performed a hierarchical cluster analysis using 31 proteins with log 2FC ≥ 2.5 (Figure 7A).

**Figure 7:**
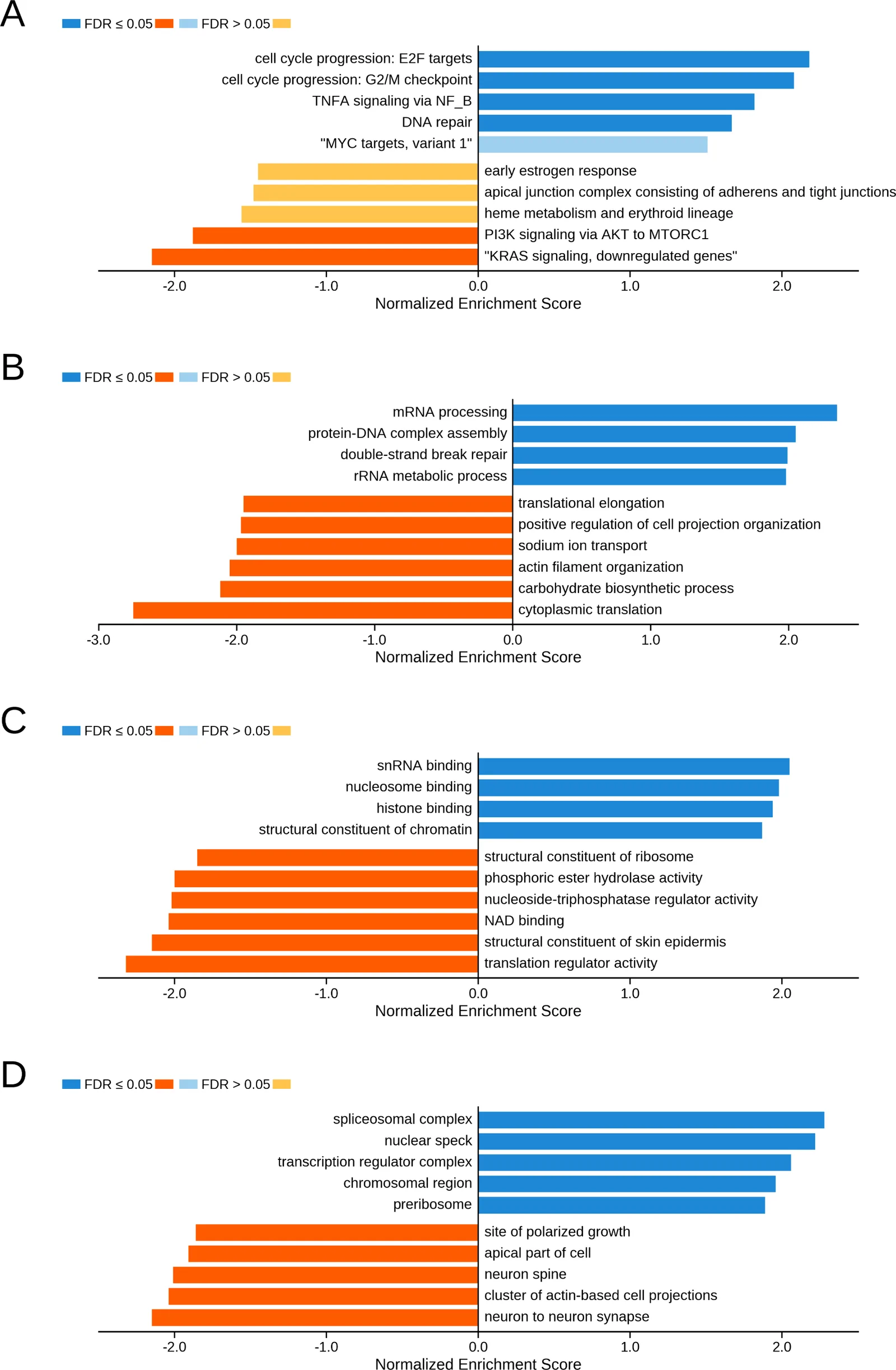
Gene set enrichment analysis resulting from proteomic analysis of lysosome enriched fraction of SKBR-3 cells treated for 48 h with T-DXd or normal human IgGs used as controls. Positive Normalized Enrichment Scores (NES) are represented by blue bars and indicate upregulation induced by T-DXd while negative NES scores (orange bars) indicate downregulation. (A) Enriched transcriptional pathway modulated by T-DXd. (B) Enriched Biological processes modulated by T-DXd. (C) Enriched Molecular Function modulated by T-DXd. (D) Enriched Cellular Component modulated by T-DXd. A color saturation scale is provided to indicate the False Discovery Ratio (FDR) associated with the modulation.

This analysis showed that on the basis of protein expression levels, the samples clustered in two distinct groups corresponding to controls and T-DXd treated samples. In particular, among the proteins upregulated in the control group we found KRT1, KRT9, KRT10, and FLG2, which are not typically reported in HER2+ breast cancer cells and likely reflect low-level keratin/skin contaminant proteins commonly detected in proteomic workflows. Conversely, among the proteins relatively enriched in T-DXd-treated samples, we detected ICAM1, SERPINA3, OLR1, and NUP160, proteins associated with inflammatory and stress-related responses. We believe that these proteomic data are in good agreement with our biochemical and morphological results reported above.

### T-DXd-Conditioned Medium Reveals Selective Cytokine Enrichment

As proteomic analyses suggested enrichment of TNFα/NF-κB signaling and inflammatory/stress-associated pathways, we explored whether conditioned media (CM) from T-DXd-treated HER2+ BCa cells displayed altered cytokine profiles. To this aim, we collected CM from various HER2+ cell lines (SKBR-3, BT474, MDA-MB-361) treated with T-DXd or IgG for 72 h and 10 days (Figure 8). Cytokine levels were measured using the Ella automated ELISA platform, focusing on interleukins (IL)-2, IL-6, IL-8, IL-10, tumor necrosis factor (TNF-α), and interferon (IFN-γ), as these are key biomarkers of inflammation.^53^ T-DXd treatment resulted in a marked and selective increase in IL-6 and IL-8 secretion across all HER2+ cell lines (Figure 8).

**Figure 8:**
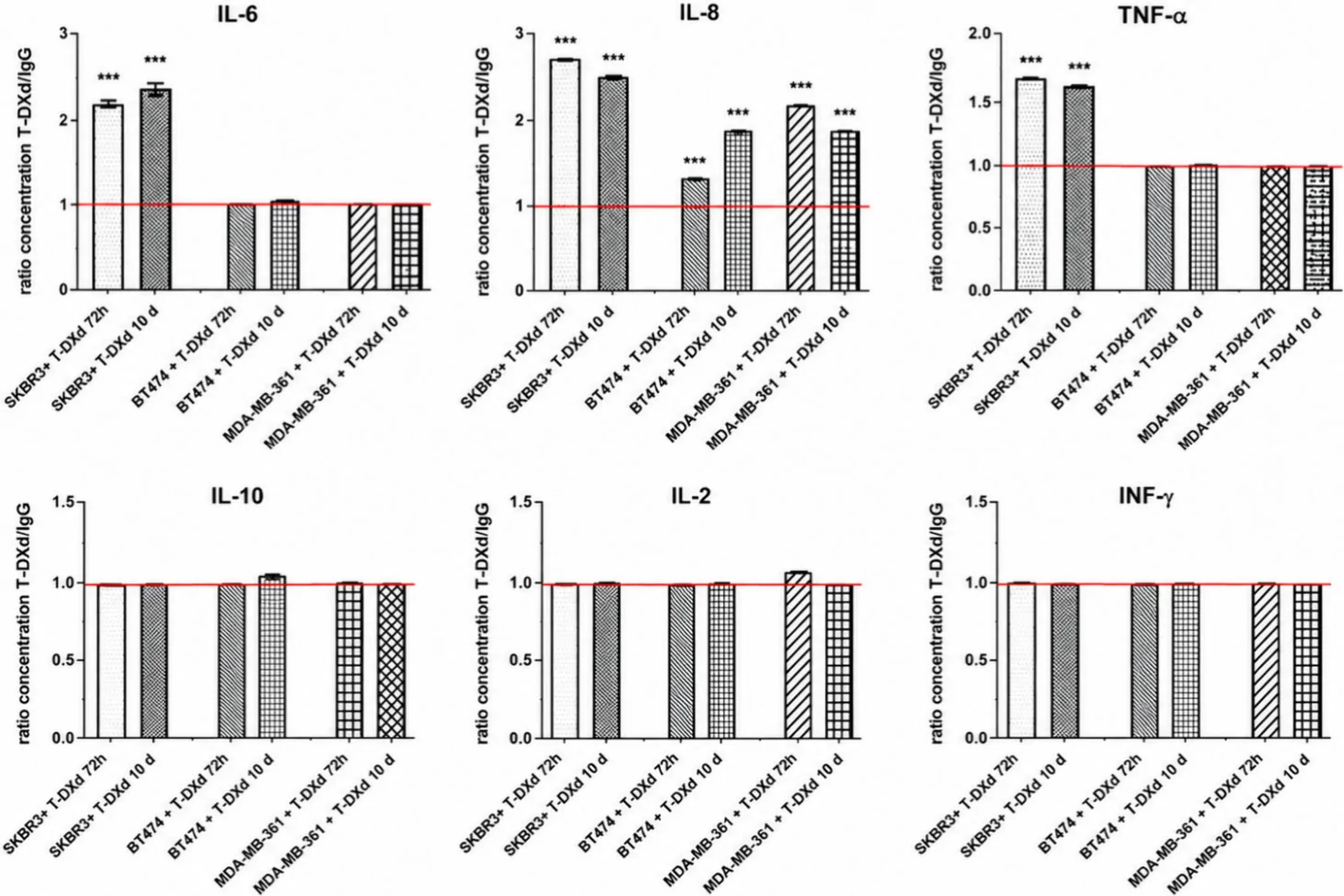
Analysis of cytokine content in the conditioned medium (CM) of T-DXd–treated breast cancer cell lines. CM was collected from HER2+ (SKBR-3, BT474, MDA-MB-361) cell lines treated with 10 μg/mL of T-DXd or control IgG for 72 h and 10 days. Cytokine concentrations (IL-2, IL-6, IL-8, IL-10, TNF-α, IFN-γ) were measured using the Ella automated ELISA platform. Concentration values were converted to a log_10_ scale, and the ratio between T-DXd and IgG treatments was calculated as log_10_(T-DXd)/log_10_(IgG). These ratios are shown on the *y*-axis, with the red line indicating the IgG reference value. Cell lines are plotted on the *x*-axis. Data are presented as median with range. Statistical significance refers to comparisons between T-DXd and IgG conditions for each cytokine and time point. IL-6 levels were significantly increased in SKBR-3 cells treated with T-DXd at both 72 h and 10 days (*p* < 0.0001). IL-8 concentrations were consistently elevated in T-DXd-treated SKBR-3 (72 h and 10 d), BT474 (72 h and 10 d), MDA-MB-361 (72 h and 10 d) (all *p* < 0.0001). TNF-α levels were also significantly higher in SKBR-3 cells at both time points (*p* < 0.0001). No significant differences were detected for IL-2, IL-10, and IFN-γ in any cell line or time point. ***p < 0.001, **p < 0.01, *p < 0.05.

Notably, similar cytokine elevations were detected in MDA-MB-231 cells, which have been classified as HER2-low. While T-DXd was originally designed to selectively target HER2-expressing cells, recent studies have shown that in HER2-low models, the cytotoxic payload can be released extracellularly through proteolytic cleavage by membrane-associated cathepsins, bypassing and/or reducing the need for receptor-mediated internalization.^17^ TNF-α was significantly elevated only in SKBR-3 cells. No changes were detected for IL-2, IL-10, or IFN-γ. These data indicate that T-DXd does not induce a general inflammatory program but rather promotes selective cytokine remodeling in a cell line–dependent manner, suggesting that these alterations may reflect the observed changes in subcellular dynamics and signaling.^54,55^ Collectively, these findings support the idea that prolonged T-DXd exposure induces tumor cell remodeling associated with with stress-related extracellular communication changes.

## Discussion

Antibody–drug conjugates (ADCs) like trastuzumab-deruxtecan (T-DXd) have revolutionized breast cancer treatment, yet the intracellular mechanisms driving their efficacy remain underexplored. Most studies focus on clinical outcomes or simplified internalization-cytotoxicity models. Here, we reveal a biphasic response to T-DXd in HER2+ cells: an early adaptive phase (2–24 h) involving slight metabolic activation, organelle contacts, and sustained HER2–ERK signaling despite limited internalization, potentially associated with trastuzumab-mediated HER2 receptor dynamics, and a later stress phase (48–72 h) characterized by lysosomal overload, mitochondrial collapse with a shift toward glycolytic activity in the absence of substantial ROS production, and nuclear/immune alterations. Further, the observed increasing in SOD2 protein levels during the later stress phase may be suggestive for an oxidative stress condition that could be scavenged by the mitochondrial antioxidant enzyme contributing to the maintenance of mitochondrial redox homeostasis. In addition, TFEB upregulation and the early reduction of LAMP1 and LC3 suggest active lysosomal-autophagic remodeling, followed at later stages by lysosomal expansion and accumulation of material potentially associated with prolonged intracellular ADC processing and payload-associated stress. These observations suggest that the intracellular response to T-DXd extends beyond the conventional view of ADC trafficking as a predominantly lysosome-centered degradative pathway leading to payload release and cell death, involving instead progressive organelle remodeling and stress adaptation. Further studies are needed to clarify the impact of T-DXd on autophagy flux. Live imaging revealed progressive HER2-associated fluorescence redistribution dynamics, peaking at 48 h, consistent with progressive engagement of the endolysosomal compartment during T-DXd exposure. Immunoelectron microscopy reveals early contact sites between HER2/T-DXd-positive lysosomes and mitochondria—a previously underexplored organelle interaction in this context. These contacts may support local energy transfer contributing to lysosomal activity during initial ADC trafficking. By 48–72 h, these events converge into the formation of a lysosome–mitochondria–nucleus triad, a spatially organized subcellular architecture not previously reported in the context of ADC treatment. This structure coincides with persistent HER2 phosphorylation, detectable pERK activity, LaminB1 redistribution, and γH2AX accumulation, suggesting nuclear stress and DNA damage. We propose that this triad may represent a lysosome-centered structural platform integrating mitochondrial and nuclear stress responses, conceptually analogous to previously described multiorganelle signaling hubs involved in coordinating organelle communication and cellular stress adaptation.^51^ This organization may reflect a spatiotemporal transition from adaptive trafficking to progressive cellular damage during prolonged ADC exposure. However, the precise functional contribution of these structures to intracellular signaling and stress propagation remains to be determined and will require dedicated mechanistic investigation in future studies. Importantly, this model suggests that T-DXd efficacy may also depend on the ability of tumor cells to engage this lysosome-driven organelle stress program. Cells that fail to undergo this coordinated transition may evade full cytotoxicity, potentially contributing to heterogeneous clinical responses. Moreover, while deruxtecan-induced DNA damage likely contributes to late-phase stress, the persistent HER2 and ERK phosphorylation observed specifically in T-DXd-treated cells suggests sustained trastuzumab-associated signaling dynamics that differ from trastuzumab alone and may involve both membrane-associated and intracellular receptor pools. This combined contribution of HER2 engagement and payload-associated stress may contribute to long-term cellular adaptation or heterogeneous therapeutic responses.

Our proteomic data are in line with a general inhibition of metabolic and biosynthetic processes and nuclear damage fostered by T-DXd as shown by our biochemical and morphological analysis, leading ultimately to apoptosis in SKBR3 and MDA-MB-361 cells. The analysis of the most deregulated proteins in our experimental model, strongly suggest that the identification of KRT1, KRT9, KRT10, and FLG2 is most likely due to a skin contamination of control samples, since they are not reported in the literature to be expressed by HER2+ cells while the upregulation of ICAM1, SERPINA3, OLR1, CEACAM1, OLR1, and NUP160 in the T-DXd treated samples, may have interesting implications. First, NUP160 is a component of the nuclear pore and its upregulation in the lysosome fraction is in good agreement with the observed derangement of the nuclear pores and cisternae found at the ultrastructural level as a consequence of T-DXd treatment. Second, other proteins in this group upregulated by T-DXd, ICAM1, SERPINA3, OLR1, and CEACAM1 are markers of inflammation. In particular, SERPINA3 is an acute-phase protein often upregulated by cytokines and its overexpression promotes tumor invasion and migration, epithelial-mesenchymal-transition, and confers resistance to cisplatin in triple-negative breast cancer cells.^56^ Our data warrant further investigation on the possibility that the overexpression of SERPINA3 in HER2+ breast cancer may have a relevant role in cancer invasion and resistance to T-DXd. Finally, upregulation of NUP160 and inflammation markers may be intertwined because depletion of NUP160 has been shown in a different model to promote autophagy and inhibit inflammation.^57^ Therefore, we suggest that NUP160 in T-DXd treated cells may contribute to the inflammation response.

The proteomic evidence of increased levels of proteins related to enhanced TNF-α signaling via NF-kB, and potentially inducing IL-6, IL-8 and TNF-α secretion in the CM, led to our finding by immunoeassay analysis that T-DXd alters the tumor cell secretome, selectively increasing IL-6 and IL-8, indeed, with no changes in IL-10, IL-2, or IFN-γ. TNF-α was elevated only in SKBR3 cells, indicating a cell line-specific inflammatory response, in agreement with previous reports linking pro-inflammatory cytokines to HER2-positive breast cancer progression.^58^ The late-stage events, including of HER2 and ERK activation, lysosomal burden, nuclear stress and cytokine release, suggests a potential involvement of the eIF2α–ATF4 axis of Integrated Stress Response (ISR). This hypothesis aligns with prior reports linking ATF4 to DNA damage and pro-inflammatory cytokine secretion.^59^ Another key observation is the sustained activation of the MAPK/ERK pathway, despite a marked reduction in total ERK protein levels. This relative persistence of phospho-ERK at later stages may have broader functional consequences beyond survival signaling. ERK activation has been implicated in the transcriptional regulation of IL-6 and IL-8 in several tumor contexts^60^ suggesting that prolonged HER2/ERK activity could shape the tumor secretome and contribute to immune microenvironment remodeling in a protumorigenic manner. Notably, sustained ERK signaling has also been linked to the upregulation of MDR1/P-glycoprotein, a known mechanism of ADC resistance.^61,62^ This effect is mediated by ERK-dependent activation of transcription factors such as AP-1 and Egr-1 and can be further reinforced by PD-L1/PD-1 axis engagement.^63,64^ From a therapeutic perspective, the persistence of HER2–ERK signaling during T-DXd treatment raises the possibility that ERK pathway inhibition could enhance ADC efficacy and delay adaptive resistance. This concept is consistent with previous studies showing that combined MAPK pathway inhibition and HER2-targeted therapies, including trastuzumab, can improve antitumor activity.^65^

In conclusion, our findings reveal that T-DXd acts not only as a cytotoxic ADC but also as a dynamic modulator of intracellular organization and organelle interactions in HER2+ breast cancer cells. T-DXd treatment promotes a progressive lysosome-associated stress phenotype characterized by mitochondrial remodeling, altered metabolic homeostasis, and increased multiorganelle interactions involving lysosomes, mitochondria, and the nuclear compartment. Together, these changes define a spatiotemporal transition from early adaptive responses toward late cellular stress and nuclear damage during prolonged ADC exposure.

Importantly, our results also indicate that HER2 engagement by trastuzumab alone contributes to early signaling rewiring, lysosomal remodeling, and metabolic adaptation but does not reproduce the broader stress-associated phenotype observed following T-DXd treatment, including increased lysosome–mitochondria–nucleus triad formation, severe metabolic impairment, and nuclear damage. This combined contribution of antibody-mediated signaling and payload-associated intracellular stress may help explain the heterogeneous responses observed across HER2+ breast cancer models. Indeed, the different HER2+ cell lines analyzed in this study displayed variable responses in HER2 downstream signaling, apoptosis induction, and cytokine secretion, consistent with previous reports describing differential sensitivity of BT474 cells to topoisomerase inhibitors.^66^

In addition, although trastuzumab-only controls were included for selected signaling, metabolic, and ultrastructural analyses, DXd-only controls were not assessed, limiting the ability to fully disentangle payload-specific effects from HER2-mediated signaling responses. Indeed, while lysosome–mitochondria–nucleus triads were consistently enriched following T-DXd treatment, their precise mechanistic contribution to intracellular signaling and stress propagation remains to be functionally validated. Finally, the cytokine remodeling observed in conditioned media was strongly cell line dependent and was not directly linked to functional immune-cell reprogramming assays.

Overall, this work provides a mechanistic framework for understanding how T-DXd reshapes intracellular architecture and organelle communication during ADC exposure and suggests that targeting ERK signaling, lysosomal function, or organelle stress adaptation pathways may represent potential strategies to enhance therapeutic efficacy and delay resistance in HER2+ breast cancer.

## Supplementary Material



## Data Availability

All data generated or analyzed during this study are included in this published article and its Supporting Information files.

## Notes

Figures S1–S4 and a table showing HER2 signaling and AKT/ERK pathway modulation; lysosomal-autophagic markers; apoptosis analyses; oxidative stress, lipid peroxidation, and antioxidant protein expression; TEM-based lysosomal remodeling and organelle-contact analyses; LAMP1 nanogold labeling; Tukey multiple-comparison statistics (PDF)

## Abbreviations

4-HNE: 4-hydroxynonenal
ACK: Ammonium-Chloride-Potassium
ADC: Antibody-Drug Conjugate
ANOVA: Analysis of Variance
BCa: Breast Cancers
CM: Conditioned Media
DAB: Diaminobenzidine
DMEM: Dulbecco’s Modified Eagle Medium
ECAR: Extracellular Acidification Rate
ER: Estrogen Receptor
FBS: Fetal Bovine Serum
FCCP: Cyanide-4-trifluoromethoxyphenylhydrazone
FCM: Flow Cytometry
FDR: false discovery ratio
FMO: Fluorescence Minus One
GSEA: gene set enrichement analysis
HEPES: 4-(2-Hydroxyethyl)-1-piperazine ethanesulfonic acid
HRP: Horseradish Peroxidase
H_2_DCFDA: 2′,7′-Dichlorodihydrofluorescein diacetate
OCR: Oxygen Consumption Rate
PBS: Phosphate Buffered Saline
PFA: Paraformaldehyde
PgR: Progesterone Receptor
ROS: Reactive Species of Oxygen
T-DXd: Trastuzumab-Deruxtecan
TME: Tumor Microenvironment
Tz: Trastuzumab
