# Supplementary material for: Intracellular Nanopharmacology of Trastuzumab Deruxtecan Reveals Lysosome-Centered Organelle Vulnerabilities in HER2-Positive Breast Cancer Cells
Source: ACS Nano Med. 2026 Jul 2;1(8):2071–90. doi: 10.1021/acsnanomed.6c00097 (PMC13565705; doi:10.1021/acsnanomed.6c00097)
Supplement: Supplementary file 1 [file nm6c00097_si_001.pdf]

Intracellular nanopharmacology of trastuzumab deruxtecan reveals lysosome-centered organelle vulnerabilities in HER2-positive breast cancer cells

Erica Tagliatti[1#], Maria Cristina Gagliani[2#], Grazia Bellese[2], Shahnaz Salamat[2], Martina Crippa[3], Manuela Sollazzo[4], Andrea Abbona[5,6], Matteo Paccagnella[5,6], Pietro Arnaldi[2,13], Lucilla Rossi[7], Andrea Petretto[7], Paola Rusmini[8], Valeria Crippa[8], Marco Carlo Merlano[9], Ornella Garrone[10], Anna Maria Porcelli[11,12], Michela Matteoli[1,13], Paola Falletta[3,14], Patrizio Castagnola[15], **Katia Cortese***[2,15]

1. *IRCCS Humanitas Research Hospital, Laboratory of Pharmacology and Brain Pathology, Rozzano,* 20072, *Milano, Italy.*
2. *DIMES, Department of Experimental Medicine, MorphoLAB, University of Genoa, 16132, Genoa, Italy.*
3. *Experimental Imaging Center, IRCCS Ospedale San Raffaele, 20132, Milan, Italy.*
4. *FABIT, Department of Pharmacy and Biotechnology, University of Bologna, 40126, Bologna, Italy.*
5. *Fondazione Arco, 12100, Cuneo, Italy*
6. *Azienda Ospedaliera Santa Croce e Carle, 12100, Cuneo, Italy*
7. *Istituto Giannina Gaslini, 16147, Genoa, Italy*
8. *Dipartimento di Scienze Farmacologiche e Biomolecolari "Rodolfo Paoletti", Dipartimento di Eccellenza 2018-2027, Università degli Studi di Milano, 20133, Milan, Italy.*
9. *Scientific Direction, Candiolo Cancer Institute, FPO-IRCCS, 10060, Candiolo, Italy.*
10. *Medical Oncology, Fondazione IRCCS Ca' Granda Ospedale Maggiore Policlinico, 20122, Milano, Italy.*
11. *FABIT, Department of Pharmacy and Biotechnology and CIRI, Interdepartmental Centre for Industrial Research 'Scienze Della Vita e Tecnologie per La Salute', University of Bologna, 40126, Bologna, Italy.*
12. *IRCCS Azienda Ospedaliero-Universitaria di Bologna, 40138, Bologna, Italy.*
13. *CNR Institute of Neuroscience, 20854, Milano, Italy.*
14. *Vita-Salute San Raffaele University, 20132, Milan, Italy.*
15. *IRCCS Azienda Ospedaliera Metropolitana, 16132, Genova, Italy.*

***Corresponding author: Katia Cortese,** cortesek@unige.it

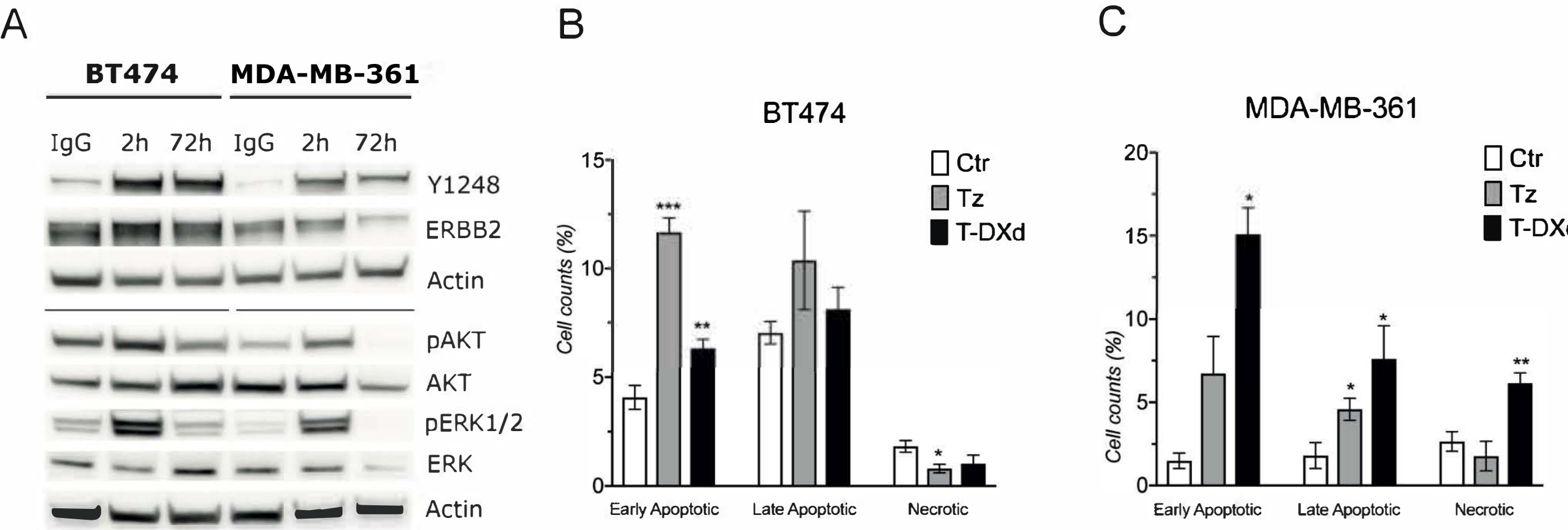

Figure S1. Time-dependent modulation of HER2 signaling, lysosomal response, and apoptosis upon T-DXd treatment in BT474 and MDA-MB-361 BCa cells. **(A)** Immunoblot analysis of HER2 phosphorylation at Y1248, total HER2 levels, and downstream signaling pathways (pAKT S473 and pERK1/2) in BT474 and MDA-MB-361 cells treated with 10 µg/ml T-DXd for 2 and 72 hours, compared to IgG control. Actin was used as a loading control. **(B,C)** Flow cytometry analysis of apoptosis in BT474 and MDA-MB-361 cells treated with T-DXd, trastuzumab, or untreated controls. The percentage of early and late apoptotic cells, and necrotic cells after 72 h of exposure to T-DXd, trastuzumab or IgG is shown. Mean values and SD (indicated as vertical bars) (n=3) are shown. Statistical significance: P <0.01 (**), P <0.001 (***), P <0.0001 (***).

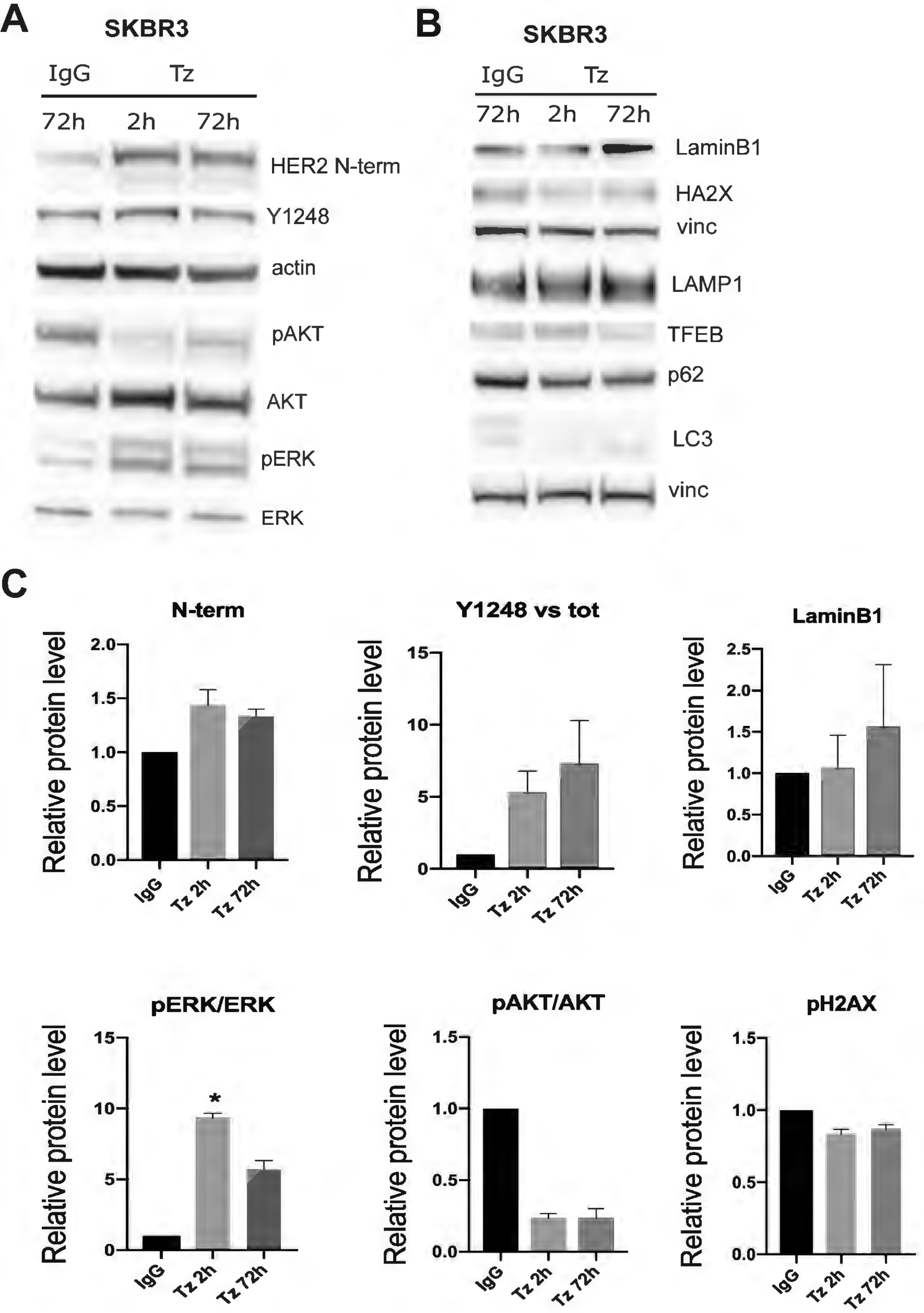

Figure S2. Modulation of HER2 signaling and lysosomal-autophagic markers following trastuzumab treatment in SKBR3 cells.

(A) Immunoblot analysis of HER2 phosphorylation at Y1248, total HER2 levels, and downstream HER2-dependent signaling pathways (pAKT S473 and pERK1/2) in SKBR3 cells treated with 10 µg/ml trastuzumab (Tz) for 2 and 72 hours, compared to IgG-treated controls. (B) Markers associated with lysosomal-autophagic remodeling (TFEB, LC3-I/II, LAMP1, SQSTM1/p62), DNA damage (γH2AX), and nuclear integrity (LaminB1) were also analyzed. Actin was used as a loading control. (C) Densitometric quantification of western blot analyses shown in panel A. Protein levels were normalized to actin and expressed relative to IgG-treated controls. Data are presented as mean ± SD (n = 3 independent experiments). Statistical analyses were performed using Kruskal–Wallis test followed by Dunn's multiple comparisons test. Statistical significance: *$P < 0.05$.

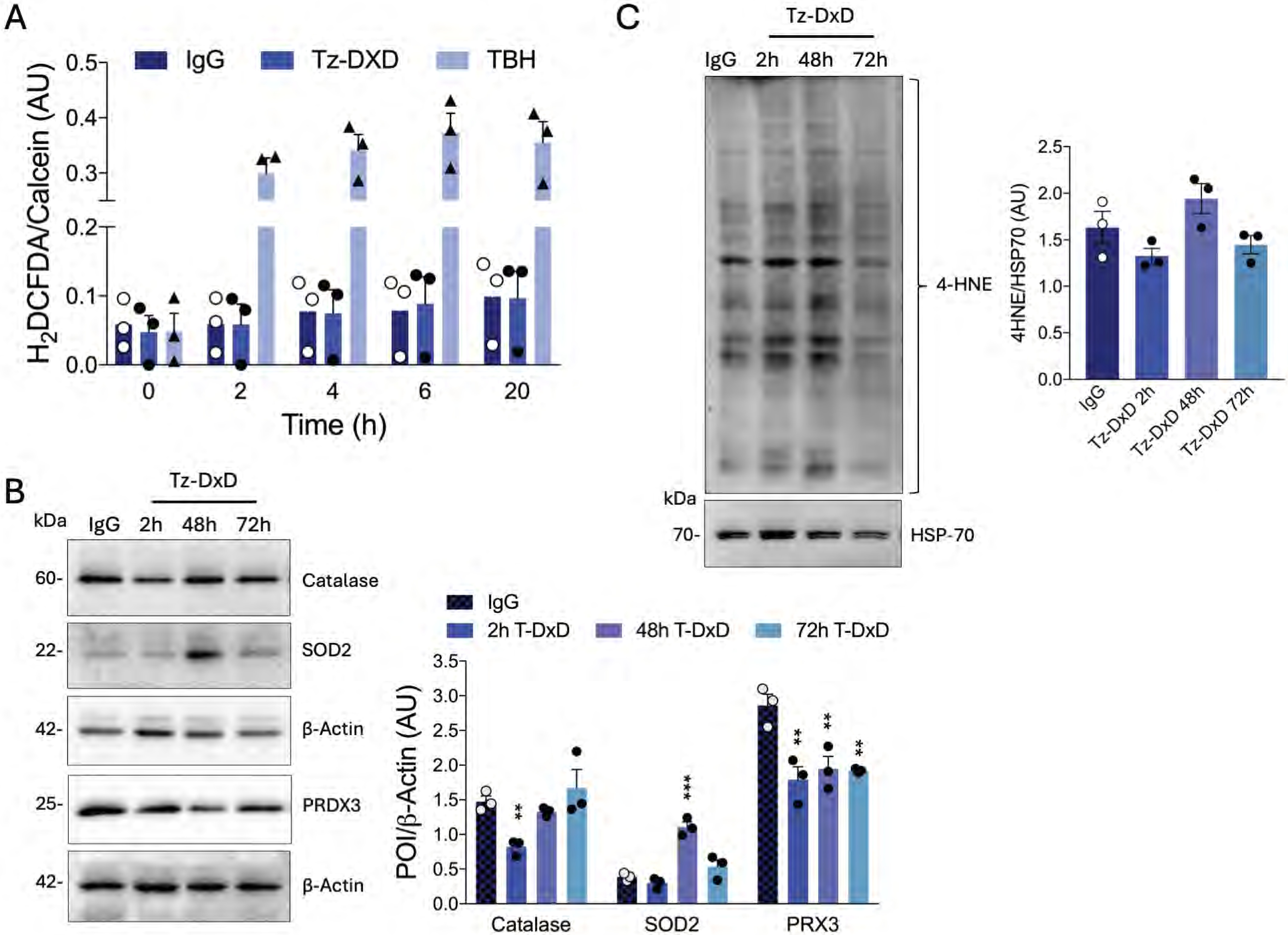

(A) H_2O_2 production in the SKBR3 cell line treated with 10 µM Tz-DXD. H_2O_2 production was measured using H_2DCFDA and data (mean ± SEM) were normalized to the number of live cells detremined by Calcein staining. Tert-Butylhydroperoxide (TBH) was used as positive control for ROS production. (B) Determination of lipids proxidation. Lipids peroxidation was analyzed by Western blot analysis of 4-HNE protein-adducts. A representative experiment is shown and densitometric data are expressed as mean ± SEM (n = 3). HSP-70 was used as loading control. (C) Antioxidant proteins levels evaluation. Catalase, SOD2 and PRDX3 antioxidant protein levels were detremined by western blot analysis. A representative experiment is shown and densitometric data are expressed as mean ± SEM (n=3). Each protein of interest (POI) was normalized to β-Actin, used as loading control. Statistical significance was calculated with unpaired Student's *t*-test ($^{**}p \leq 0.01$; $^{***}p \leq 0.001$).

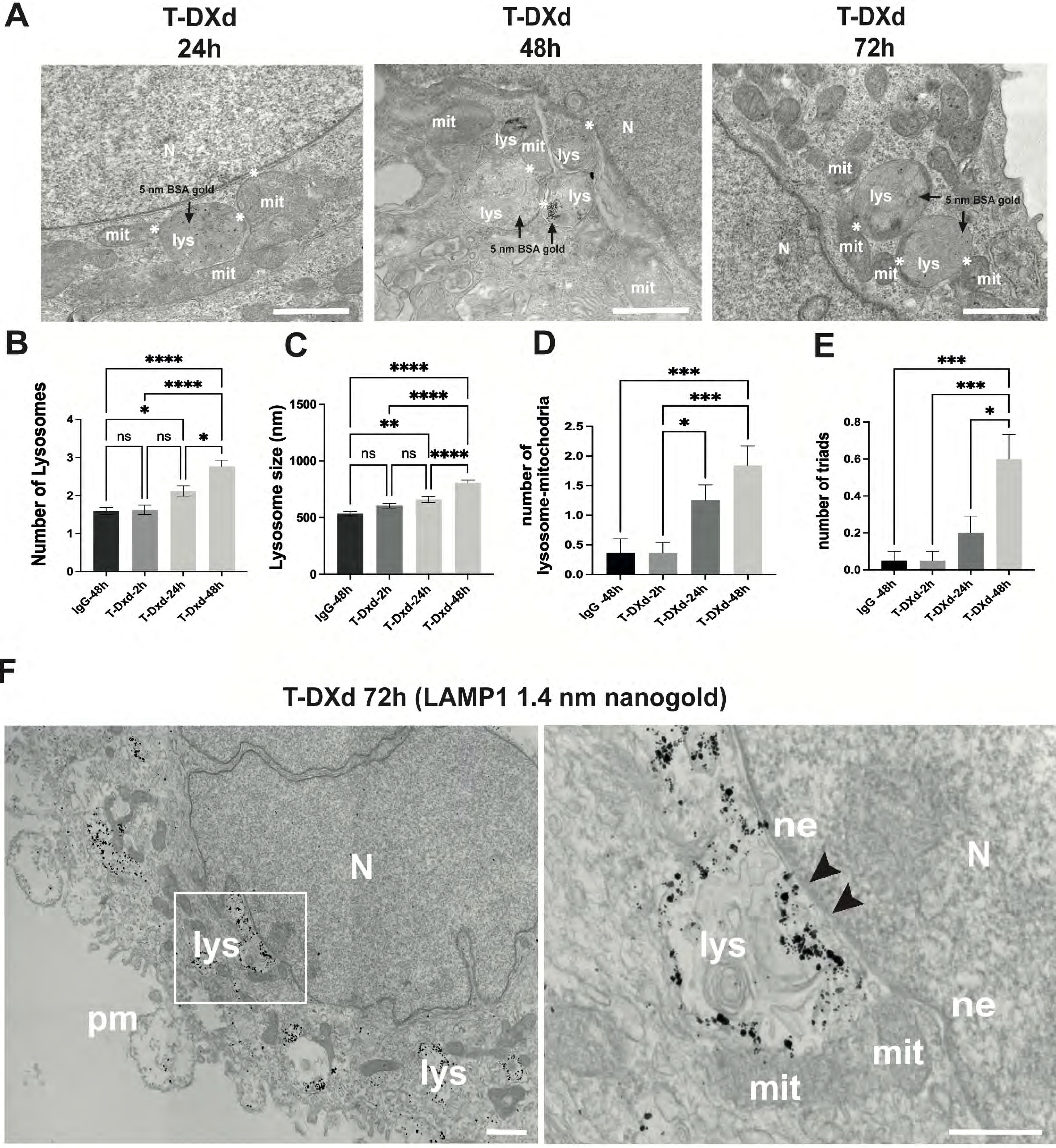

Figure S4. Ultrastructural analysis of lysosomal remodeling and nanoscale organelle contacts in T-DXd–treated cells. (A) Transmission electron microscopy (TEM) images of SKBR3 cells showing lysosomes identified by internalized 5 nm BSA–gold nanoparticles (electron-dense particles) at 24, 48, and 72 h following T-DXd treatment. (B–E) Quantitative morphometric analysis of lysosomal and organelle contact parameters: (B) number of lysosomes per cell, (C) average lysosomal size, (D) number of lysosome–mitochondria contacts per cell, and (E) number of lysosome–mitochondria–nucleus triads per cell. Lysosomes were identified based on the presence of internalized BSA–gold particles. Organelle contacts were defined as membrane appositions with an inter-organelle distance ≤ 30 nm. Quantification was performed on 30 TEM images per condition randomly acquired at 25,000× magnification from three independent experiments (n = 3). Data are presented as mean ± SEM. Statistical analysis was performed using one-way ANOVA followed by Tukey's multiple comparisons test. Significance levels were defined as $P < 0.05$ (*), $P < 0.01$ (**), $P < 0.001$ (***), and $P < 0.0001$ (****). (F) **Nanogold labeling of lysosomes.** 1.4 nm nanogold labeling of LAMP1 in T-DXd-treated cells, highlighting LAMP1-positive lysosomes near the nuclear envelope and mitochondria (white box, enlargement shown in the right panel). The nuclear envelope exhibits structural alterations in response to T-DXd treatment. Scale bars: 1 μm, 500 nm.

Supplementary table 1

Compare cell means regardless of rows and columns								
Number of families	1							
Number of comparisons per family	36							
Alpha	0,05							
Tukey's multiple comparisons test	Mean Diff.	95.00% CI of diff.	Significant?	Summary	Adjusted P Value			
24:NT vs. 24:IgG	0,1313	0.05777 to 0.2047	Yes	****	<0.0001			
24:NT vs. 24:compound	-0,02800	-0.1015 to 0.04548	No	ns	0,9281			
24:NT vs. 48:NT	-0,03075	-0.1042 to 0.04273	No	ns	0,8850			
24:NT vs. 48:IgG	0,07250	-0.0009800 to 0.1460	No	ns	0,0552			
24:NT vs. 48:compound	-0,7313	-0.8047 to -0.6578	Yes	****	<0.0001			
24:NT vs. 72:NT	0,1043	0.03077 to 0.1777	Yes	**	0,0016			
24:NT vs. 72:IgG	0,1735	0.1000 to 0.2470	Yes	****	<0.0001			
24:NT vs. 72:compound	-0,8153	-0.8887 to -0.7418	Yes	****	<0.0001			
24:IgG vs. 24:compound	-0,1593	-0.2327 to -0.08577	Yes	****	<0.0001			
24:IgG vs. 48:NT	-0,1620	-0.2355 to -0.08852	Yes	****	<0.0001			
24:IgG vs. 48:IgG	-0,05875	-0.1322 to 0.01473	No	ns	0,1981			
24:IgG vs. 48:compound	-0,8625	-0.9360 to -0.7890	Yes	****	<0.0001			
24:IgG vs. 72:NT	-0,02700	-0.1005 to 0.04648	No	ns	0,9407			
24:IgG vs. 72:IgG	0,04225	-0.03123 to 0.1157	No	ns	0,5975			
24:IgG vs. 72:compound	-0,9465	-1.020 to -0.8730	Yes	****	<0.0001			
24:compound vs. 48:NT	-0,002750	-0.07623 to 0.07073	No	ns	>0.9999			
24:compound vs. 48:IgG	0,1005	0.02702 to 0.1740	Yes	**	0,0025			
24:compound vs. 48:compound	-0,7033	-0.7767 to -0.6298	Yes	****	<0.0001			
24:compound vs. 72:NT	0,1323	0.05877 to 0.2057	Yes	****	<0.0001			
24:compound vs. 72:IgG	0,2015	0.1280 to 0.2750	Yes	****	<0.0001			
24:compound vs. 72:compound	-0,7873	-0.8607 to -0.7138	Yes	****	<0.0001			
48:NT vs. 48:IgG	0,1033	0.02977 to 0.1767	Yes	**	0,0018			
48:NT vs. 48:compound	-0,7005	-0.7740 to -0.6270	Yes	****	<0.0001			
48:NT vs. 72:NT	0,1350	0.06152 to 0.2085	Yes	****	<0.0001			
48:NT vs. 72:IgG	0,2043	0.1308 to 0.2777	Yes	****	<0.0001			
48:NT vs. 72:compound	-0,7845	-0.8580 to -0.7110	Yes	****	<0.0001			
48:IgG vs. 48:compound	-0,8038	-0.8772 to -0.7303	Yes	****	<0.0001			
48:IgG vs. 72:NT	0,03175	-0.04173 to 0.1052	No	ns	0,8663			
48:IgG vs. 72:IgG	0,1010	0.02752 to 0.1745	Yes	**	0,0023			
48:IgG vs. 72:compound	-0,8878	-0.9612 to -0.8143	Yes	****	<0.0001			
48:compound vs. 72:NT	0,8355	0.7620 to 0.9090	Yes	****	<0.0001			
48:compound vs. 72:IgG	0,9048	0.8313 to 0.9782	Yes	****	<0.0001			
48:compound vs. 72:compound	-0,08400	-0.1575 to -0.01052	Yes	*	0,0163			
72:NT vs. 72:IgG	0,06925	-0.004230 to 0.1427	No	ns	0,0763			
72:NT vs. 72:compound	-0,9195	-0.9930 to -0.8460	Yes	****	<0.0001			
72:IgG vs. 72:compound	-0,9888	-1.062 to -0.9153	Yes	****	<0.0001			
Test details	Mean 1	Mean 2	Mean Diff.	SE of diff.	N1	N2	q	DF
24:NT vs. 24:IgG	1,000	0,8688	0,1313	0,02184	4	4	8,499	27,00
24:NT vs. 24:compound	1,000	1,028	-0,02800	0,02184	4	4	1,813	27,00
24:NT vs. 48:NT	1,000	1,031	-0,03075	0,02184	4	4	1,991	27,00
24:NT vs. 48:IgG	1,000	0,9275	0,07250	0,02184	4	4	4,695	27,00
24:NT vs. 48:compound	1,000	1,731	-0,7313	0,02184	4	4	47,35	27,00
24:NT vs. 72:NT	1,000	0,8958	0,1043	0,02184	4	4	6,751	27,00

**24:NT vs. 72:IgG**	1,000	0,8265	0,1735	0,02184	4	4	11,24	27,00
**24:NT vs. 72:compound**	1,000	1,815	-0,8153	0,02184	4	4	52,79	27,00
**24:IgG vs. 24:compound**	0,8688	1,028	-0,1593	0,02184	4	4	10,31	27,00
**24:IgG vs. 48:NT**	0,8688	1,031	-0,1620	0,02184	4	4	10,49	27,00
**24:IgG vs. 48:IgG**	0,8688	0,9275	-0,05875	0,02184	4	4	3,805	27,00
**24:IgG vs. 48:compound**	0,8688	1,731	-0,8625	0,02184	4	4	55,85	27,00
**24:IgG vs. 72:NT**	0,8688	0,8958	-0,02700	0,02184	4	4	1,748	27,00
**24:IgG vs. 72:IgG**	0,8688	0,8265	0,04225	0,02184	4	4	2,736	27,00
**24:IgG vs. 72:compound**	0,8688	1,815	-0,9465	0,02184	4	4	61,29	27,00
**24:compound vs. 48:NT**	1,028	1,031	-0,002750	0,02184	4	4	0,1781	27,00
**24:compound vs. 48:IgG**	1,028	0,9275	0,1005	0,02184	4	4	6,508	27,00
**24:compound vs. 48:compound**	1,028	1,731	-0,7033	0,02184	4	4	45,54	27,00
**24:compound vs. 72:NT**	1,028	0,8958	0,1323	0,02184	4	4	8,564	27,00
**24:compound vs. 72:IgG**	1,028	0,8265	0,2015	0,02184	4	4	13,05	27,00
**24:compound vs. 72:compound**	1,028	1,815	-0,7873	0,02184	4	4	50,98	27,00
**48:NT vs. 48:IgG**	1,031	0,9275	0,1033	0,02184	4	4	6,686	27,00
**48:NT vs. 48:compound**	1,031	1,731	-0,7005	0,02184	4	4	45,36	27,00
**48:NT vs. 72:NT**	1,031	0,8958	0,1350	0,02184	4	4	8,742	27,00
**48:NT vs. 72:IgG**	1,031	0,8265	0,2043	0,02184	4	4	13,23	27,00
**48:NT vs. 72:compound**	1,031	1,815	-0,7845	0,02184	4	4	50,80	27,00
**48:IgG vs. 48:compound**	0,9275	1,731	-0,8038	0,02184	4	4	52,05	27,00
**48:IgG vs. 72:NT**	0,9275	0,8958	0,03175	0,02184	4	4	2,056	27,00
**48:IgG vs. 72:IgG**	0,9275	0,8265	0,1010	0,02184	4	4	6,541	27,00
**48:IgG vs. 72:compound**	0,9275	1,815	-0,8878	0,02184	4	4	57,49	27,00
**48:compound vs. 72:NT**	1,731	0,8958	0,8355	0,02184	4	4	54,11	27,00
**48:compound vs. 72:IgG**	1,731	0,8265	0,9048	0,02184	4	4	58,59	27,00
**48:compound vs. 72:compound**	1,731	1,815	-0,08400	0,02184	4	4	5,440	27,00
**72:NT vs. 72:IgG**	0,8958	0,8265	0,06925	0,02184	4	4	4,484	27,00
**72:NT vs. 72:compound**	0,8958	1,815	-0,9195	0,02184	4	4	59,54	27,00
**72:IgG vs. 72:compound**	0,8265	1,815	-0,9888	0,02184	4	4	64,03	27,00
